# Poly-ε-Caprolactone/Halloysite Nanotube Composites for Resorbable Scaffolds: Effect of Processing Technology on Homogeneity and Electrospinning

**DOI:** 10.3390/polym13213772

**Published:** 2021-10-31

**Authors:** Muriel Józó, Róbert Várdai, Nóra Hegyesi, János Móczó, Béla Pukánszky

**Affiliations:** 1 Laboratory of Plastics and Rubber Technology, Department of Physical Chemistry and Materials Science, Budapest University of Technology and Economics, P.O. Box 91, H-1521 Budapest, Hungary; jozo.muriel@vbk.bme.hu (M.J.); vardai.robert@vbk.bme.hu (R.V.); hegyesi.nora@vbk.bme.hu (N.H.); jmoczo@edu.bme.hu (J.M.); 2 Institute of Materials and Environmental Chemistry, Research Centre for Natural Sciences, ELKH Eötvös Loránd Research Network, P.O. Box 286, H-1519 Budapest, Hungary

**Keywords:** homogenization, mixing, compression, film casting, aggregation, thermal analysis, mechanical properties

## Abstract

Polycaprolactone (PCL)/halloysite composites were prepared to compare the effect of homogenization technology on the structure and properties of the composites. Halloysite content changed from 0 to 10 vol% in six steps and homogeneity was characterized by various direct and indirect methods. The results showed that the extent of aggregation depends on technology and on halloysite content; the size and number of aggregates increase with increasing halloysite content. Melt mixing results in more homogeneous composites than the simple compression of the component powders or homogenization in solution and film casting. Homogeneity and the extent of aggregation determines all properties, including functionality. The mechanical properties of the polymer deteriorate with increasing aggregation; even stiffness depends on homogeneity. Strength and deformability decreases drastically as the number and size of aggregates increase. Not only dispersed structure, but also the physical state and crystalline structure of the polymer influence homogeneity and properties. The presence of the filler affects the preparation of electrospun fiber scaffolds as well. A part of the filler is excluded from the fibers while another part forms aggregates that complicates fiber spinning and deteriorates properties. The results indicate that spinning is easier and the quality of the fibers is better if a material homogenized previously by melt mixing is used for the production of the fibers.

## 1. Introduction

The interest in bioplastics has increased enormously in recent years. They are used in a wide range of applications including packaging, agriculture, and various consumer goods, but they are also used as technical parts in the automotive or machine industries [1,2,3]. Biopolymers can be used as materials with high added value also in biomedicine as sutures, implants, scaffolds, and as carriers for controlled drug delivery [4,5,6]. These polymers would be especially suitable for the production of resorbable polymeric scaffolds serving as temporary extracellular matrices for tissue regeneration [7]. However, the basic condition for the use of biopolymers in such applications is that the rate of tissue regeneration and the degradation of the scaffold should match each other [8]. Moreover, the applied polymer must be biocompatible and hazardous metabolites cannot form during its degradation [9].

Poly-ε-caprolactone (PCL) is a biopolymer that is applied in considerable amounts in biomedicine [10,11]. It is often used as matrix material for drug delivery systems as well as for the production of scaffolds with or without an active component. However, because its degradation is very slow, requiring several years, PCL cannot be used as resorbable scaffold [12]. PCL, being a polyester, degrades through the cleavage of its ester groups. Its hydrolytic degradation is very slow at neutral pH, proceeding at a proper rate only under strongly acidic or alkali conditions. However, such conditions do not occur in the application in question. A much more promising way to control the degradation of polyesters is the use of enzymatic catalysis, which may take place under physiologic conditions. Lipases and esterases catalyze the degradation of PCL in both solvents and water [13,14]. However, scaffolds are implanted into the body of the patient and the appropriate enzyme cannot access the polymer to catalyze degradation. Consequently, it must be incorporated into the polymer to achieve controlled degradation and the resorption of the scaffold.

In a previous study, a lipase enzyme was successfully adsorbed on the surface of halloysite nanotubes to prepare a device, which was then incorporated into poly-ε-caprolactone [15]. The supported enzyme was mixed with the polymer, and compression molded films were prepared at 70 °C. The supported enzyme degraded PCL efficiently, and the rate of degradation depended on the amount of enzyme adsorbed. This was the first time when the enzyme catalyzing degradation was not dissolved in the degradation medium, but in the polymer, thus allowing the preparation of a resorbable scaffold with a controlled lifetime. However, the technology used for the production of the composite containing the supported enzyme was inadequate, the material was inhomogeneous and thus degradation proceeded in an inhomogeneous way as well. Techniques leading to a more homogeneous material had to be found in order to prove the viability of the approach.

The electrospinning technique has received considerable attention lately because it is an affordable, cost-effective, and easy to use technology for creating nanofibrous scaffolds for tissue engineering purposes, as well as drug delivery systems and wound dressings [16,17,18,19]. Electrospun drug delivery systems have numerous advantages, such as large loading capacity [20], the wide selection of polymers depending on the compatibility of the other ingredients of the formulation, simple and cost-effective process [21], and the possibility to regulate drug delivery [22,23]. Other techniques, like additive manufacturing, plastic processing techniques, casting, etc., do not have all these advantages.

Nanofillers, including halloysite nanotubes, can be applied in functional applications, among others as supports for active components like an enzyme used as catalyst in the controlled degradation of PCL [24,25,26,27,28]. The main problem usually encountered during the preparation of nanocomposites is homogeneity. Small particles tend to aggregate, leading to inferior mechanical properties, functional characteristics, and aesthetics [29,30,31]. The aggregation of the filler depends on its size, surface energy, and the conditions of homogenization [32,33,34]. Strong adhesion of the particles results in a larger extent of aggregation, which can be compensated by shear forces separating the particles [35,36]. However, shear forces cannot be increased to an unlimited extent because of technological reasons (limitations of the equipment, viscosity of the medium) or because of the degradation of the material. Accordingly, the selection of the appropriate conditions is extremely important to ensure homogeneity and thus the quality of the material. The degree of aggregation can also be decreased by the use of interfacial agents, which decrease the surface energy of the nanofiller [37,38,39]. However, such a surface coating would hinder or completely prevent the adsorption of the enzyme used for catalysis that is the goal and essence of the approach used.

Using the experience obtained in our previous study [15], the goal of this project was to investigate the effect of processing technology on the homogeneity of PCL/halloysite nanotube composites. Three technologies were selected for the preparation of the composites. The first was the one used before, which comprised the grinding of the polymer into powder, mixing it with the halloysite, and compression molding it into films. The support was homogenized with the polymer in an internal mixer in the second technique. Scaffolds are often produced by electrospinning from solution; thus, the third method was the homogenization of the tubes with the polymer in solution and the casting of films for comparative purposes. Finally, electrospun fibers have been also prepared in order to study the effect of nanotubes on the spinning process and the quality of the fibers. The incorporation of the filler into the polymer, homogeneity, and the structure and property of the composites were studied by various methods.

## 2. Materials and Methods

The poly-ε-caprolactone used in the experiments was the Capa 6800 grade produced by Perstorp Holding AB, Malmö, Sweden. According to the producer, its molecular mass is 80 kDa, MFR 2–4 g/min (160 °C, 5 kg), and its melting temperature is 58–60 °C. The halloysite used as support was the New Zealand CC Ultrafine H filler obtained from Imerys Ceramics, Paris, France. The specific surface area of the filler was determined by BET nitrogen adsorption as 27 m^2^/g (Quantachrome Nova 2000, Anton Paar GmbH, Graz, Austria). The length of the tubes is between 0.4 and 2.5 µm, and their outer and inner diameter are 100 and 20–50 nm (TEM, Tecnai G2 Spirit, FEI, Thermo Fisher, Waltham, MA, USA), respectively. Particle size and its distribution was determined by laser light scattering (Horiba LA 950 A2, Loos, France). The solvent used for the casting of films was dichloromethane (DCM) obtained from Molar Chemical Kft., Halásztelek, Hungary. DCM is stabilized with ethanol; its density is 1.33 g/cm^3^.

Before homogenization the filler was dried in a vacuum oven (Memmert VO400, Memmert GmbH, Büchenbach, Germany) at 25 °C for 24 h. According to the first technique, the halloysite nanotubes and the polymer were homogenized in an internal mixer (Haake Rhecord EV 10V, Haake Technik GmbH, Vreden, Germany). The components were homogenized at 70 °C and 30 rpm for 10 min. Subsequently, the homogenized material was compression molded (Fontijne SRA 100, Fontijne Presses, Delft, The Netherlands) into 1 mm thick plates at 70 °C and 150 kN force.

Compression molded samples were prepared the same way as in the previous study [15]. PCL granules were cooled with liquid nitrogen and then ground to powder. The halloysite and the polymer powder were homogenized by mixing with a spatula and vigorous shaking. The powder mixture was compression molded into films of 200 µm thickness at 70 °C and 150 kN force in about 5 min using a Fontijne SRA 100 (Fontijne Presses, Delft, The Netherlands) apparatus.

A DCM solution containing 5 wt% PCL was prepared for film casting. Halloysite was added after the complete dissolution of the polymer, the solution was sonicated for 5 min, and then it was shaken for 5 min at 200 rpm. Sonication and shaking were repeated twice and then the solution was poured into Teflon crystallization cups in several steps until the 200 µm thickness was achieved. The composite samples prepared with all three technologies contained 0, 1, 3, 5, 7, and 10 vol% halloysite nanotubes.

PCL fibers containing various amounts of halloysite nanotubes were prepared by electrospinning (Spincube, SpinSplit Kft., Budapest, Hungary) at ambient temperature, 15 kV voltage, 15 cm collector distance, and a feeding rate of 2 µL/s. The solvent used was DCM, and the solution contained 10 wt% PCL, as well as various amounts of halloysite.

The actual halloysite content of the samples was determined by thermogravimetric analysis (TGA) using a Perkin Elmer TGA 6 (Perkin Elmer Inc., Waltham, MA, USA) apparatus. A quantity of 10–15 mg of each sample was heated from 30 to 700 °C and then held at this temperature for 10 more min in O_2_ atmosphere. The measurements were done in triplicate.

The homogeneity and the structure of the samples were studied by scanning electron microscopy (SEM) (Jeol JSM 6380 LA, Jeol, Tokyo, Japan). Micrographs were recorded on fracture surfaces created at liquid nitrogen temperature. SEM was used to study the morphology of electrospun fibers as well. These latter were investigated also by digital optical microscopy using a Keyence VHX 5000 apparatus (Keyence Corporation, Osaka, Japan).

Crystalline structure as well as the thermal characteristics of the composites were studied by differential scanning calorimetry (DSC) and dynamic mechanical thermal analysis (DMTA). DSC measurements were carried out using a Perkin Elmer Diamond DSC apparatus (Perkin Elmer Inc., Waltham, MA, USA) on 3–5 mg samples in two heating runs and a cooling run. First, the samples were cooled to −100 °C, kept there for 3 min, then they were heated up to 100 °C at 10 °C/min rate, kept at this temperature for 3 min, cooled down to −100 °C at the same rate, and after 3 min holding time heated up again as before. Dynamic mechanical thermal analysis (DMTA) was carried out using a Perkin Elmer Diamond DMA (Perkin Elmer Inc., Waltham, MA, USA) equipment. Samples of 50 mm long and 5 mm wide with the thickness of the plate (1 mm) or the film (200 µm) were cut for the tests. The measurements were done in the temperature range of −100 and 55 °C at 1 Hz frequency and 2 °C/min heating rate.

Specimens corresponding to the ISO 527 standard (type 5A) were cut from the plates or films and tensile properties were determined using a Zwick/Roell 1445 tensile testing machine (Zwick, Roell Group, Ulm, Germany). Gauge length was 45 mm and cross-head speed 10 mm/min. Young’s modulus, yield stress and yield strain, as well as tensile strength and elongation-at-break were calculated from measured force vs. elongation traces. The mechanical properties of electrospun fiber mats were also characterized by tensile testing using an Instron 34SC-05 instrument (Illinois Tool Works Inc., Norwood, MA, USA). Mats with approximately 300 mg weight were compressed slightly into specimens of 10 × 50 mm dimensions and then tested at 20 mm gauge length and 5 mm/min cross-head speed.

## 3. Results

The results are presented in several sections. The actual halloysite content of the composites prepared and the homogeneity of the filler in the polymer are discussed in the first two. Thermal analysis and crystalline structure are presented subsequently, followed by the discussion of mechanical properties and the effect of homogeneity on it. The structure of electrospun fibers and the mechanical properties of fiber mats prepared from them are shown in the last section together with a brief note on the practical relevance of the results.

### 3.1. Halloysite Content

**Figure 1:**
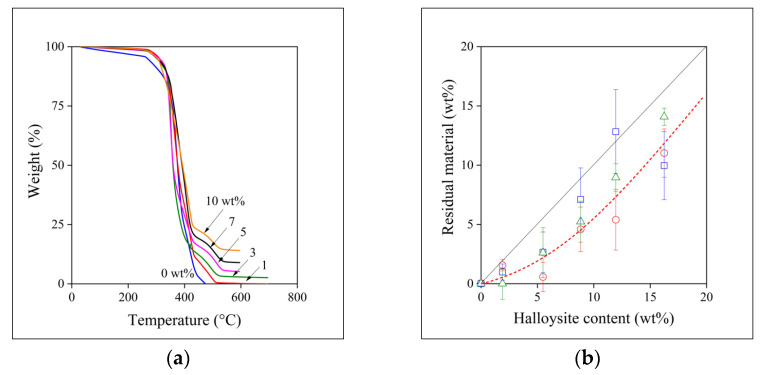
Determination of the halloysite content of PCL/halloysite composites by TGA measurements. (**a**) Weight loss of the material during the measurement. Halloysite content increases from bottom to top. (**b**) Correlation between the nominal and the measured (residual material) halloysite content of the composites. Symbols: (○) film, (☐) compressed, (△) melt mixed. The straight line indicates the equality of the two quantities.

One would expect that filler content corresponds to the nominal value and does not change during the homogenization process. However, this rarely happens because of the technical difficulties encountered practically in all technologies. The different size and density of the components usually lead to segregation already at the preliminary stage of mixing and further loss may occur during the homogenization process itself as well as in the final phase of sample preparation. Accordingly, the actual filler content of the composites was checked by thermal gravimetric (TGA) measurements (Tables S5–S7, Figures S15–S27). The results of the series of measurements done on the materials prepared by melt mixing in the internal mixer is presented in Figure 1a as an example. The results apparently correspond to the expectations. The halloysite nanotubes have only a slight influence on the decomposition of the polymer, the onset temperature of decomposition changes between 264 and 272 °C without any tendency in the case of the samples homogenized in the internal mixer, and the amount of residual material increases with nominal filler content. One would expect a good agreement between the nominal and the actual filler content as a result.

The two quantities, i.e., the amount of filler added to the polymer at the beginning of the homogenization process (halloysite content) and the actually measured one determined by TGA analysis (residual material), are plotted against each other in Figure 1b. Equal amounts of the two quantities is indicated by the straight line in the figure. We must call the attention here to the fact that the correlation drawn in this and in all other figures is not fitted by a model, but it is a line to guide the eye. The discrepancy is clear, a systematic difference can be observed between the nominal and the actual filler content. The scatter of the points is considerable, indicating the difficulties encountered during composite preparation. The difference as well as the scatter varies with the technology of sample preparation and it is quite large for film casting and especially for the compressed samples. The most consistent results were obtained for the samples homogenized in the internal mixer. The differences between the nominal and actual values decrease slightly with increasing filler content in this latter case indicating that absolute amounts also play a role, the homogenization of a small amount of filler with the polymer is difficult, relative losses are larger. Obviously, losses during homogenization must be minimized, but definitely considered during the preparation of the composites or any device containing such nanofillers.

### 3.2. Homogeneity, Distribution

**Figure 2:**
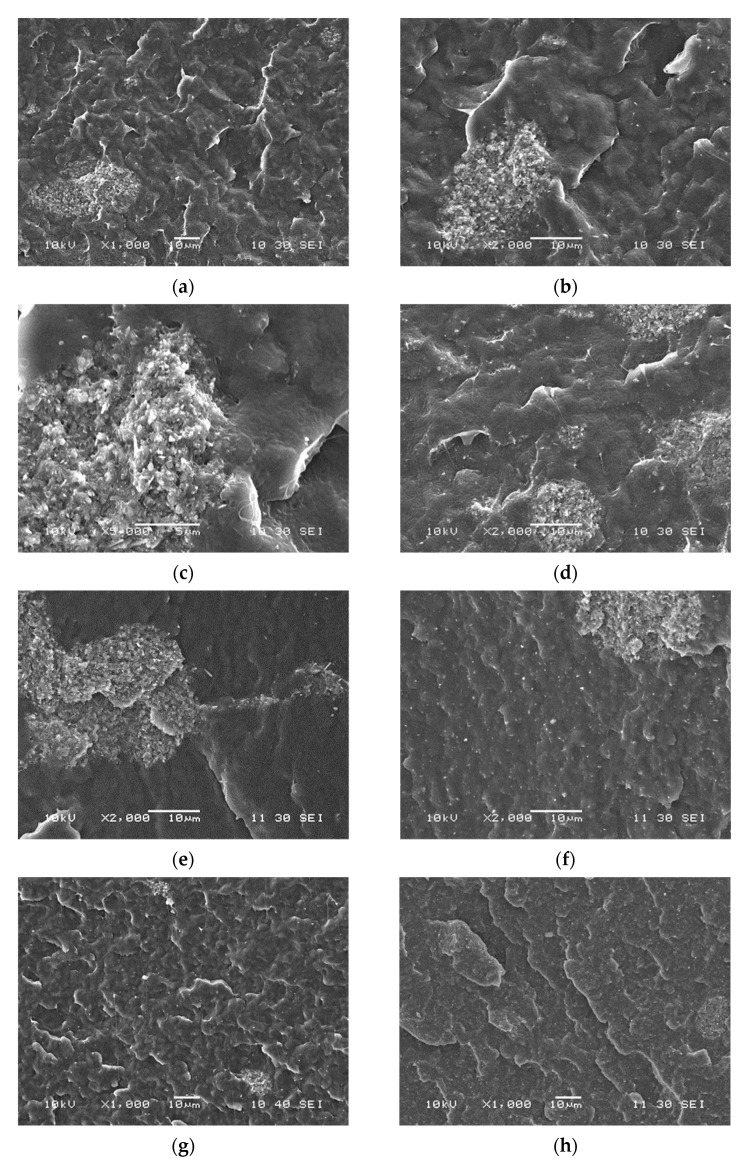

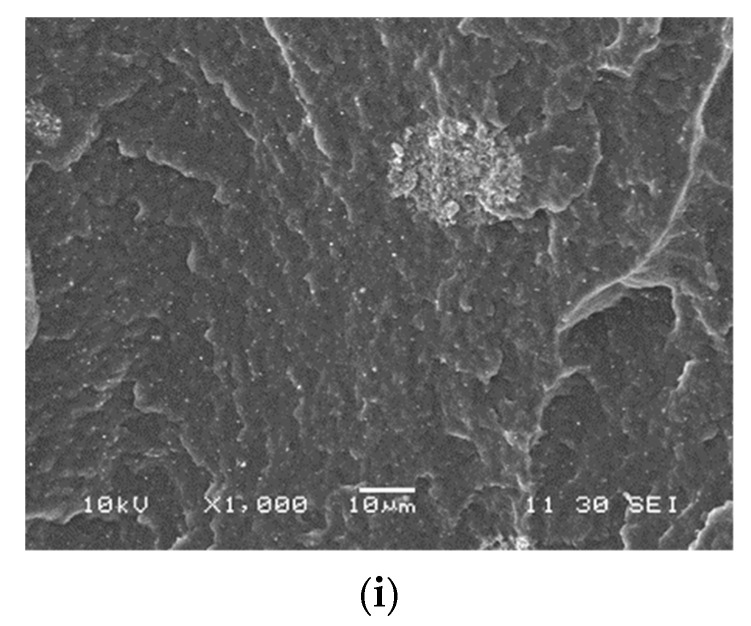
Detection and characterization of aggregation in PCL/halloysite composites by scanning electron microscopy. Halloysite content, technology, and magnification: (**a**) 5 vol%, film, 1000×; (**b**) 5 vol%, film, 2000×; (**c**) 5 vol%, film, 5000×; (**d**) 5 vol%, film, 2000×; (**e**) 5 vol%, compressed, 2000×; (**f**) 5 vol%, melt mixed, 2000×; (**g**) 1 vol% melt mixed, 1000×; (**h**) 5 vol%, melt mixed, 1000×; (**i**) 10 vol%, melt mixed, 1000×.

Homogeneity is even more important than the actual filler content especially in the application in question, i.e., when the filler is used as support for an enzyme to prepare a device for the controlled degradation of the polymer. However, as noted before, fillers with high surface energy and small size tend to aggregate, so their homogenization is difficult. The homogeneity of the composites prepared in this study was checked directly by microscopy, but also by indirect methods through the determination of the composition dependence of properties. SEM micrographs recorded on the surface of crio-fractured samples are shown in Figure 2. The first three micrographs were recorded on films at different magnifications (Figure 2a–c). A relatively large aggregate with the size of 10–20 µm is clearly seen in the bottom left corner of the micrographs. The micrographs show that the aggregate consists of a large number of smaller particles. The second series of micrographs (Figure 2d–f) demonstrates the effect of technology on homogeneity, or more exactly on inhomogeneity, aggregation. The number of aggregates is quite large in the cast film (Figure 2d), a large aggregate can be observed in the compressed plate (Figure 2e), whereas the structure of the melt mixed sample seems to be more homogeneous than that of the other two (Figure 2f). The effect of composition on structure is shown in Figure 2g–i for the samples homogenized by melt mixing. The number and size of aggregates seem to increase with increasing filler content. However, one must be extremely cautious drawing conclusions about the overall structure of any material from SEM micrographs, as the technique shows only a very small area within the material. Further analysis is needed to estimate the overall homogeneity of the materials prepared.

**Figure 3:**
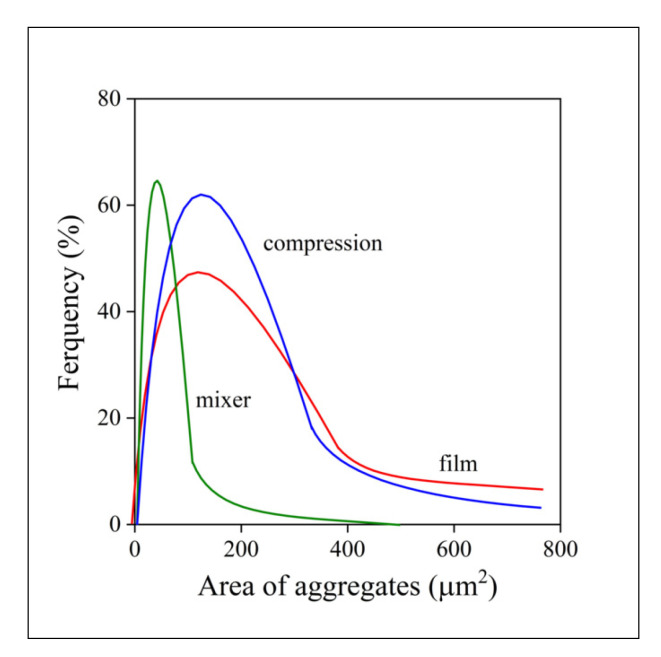
Size distribution of aggregates in PCL/halloysite composites at 5 vol% filler content. Effect of homogenization technology.

Accordingly, the number and size distribution of the aggregates were also determined by the image analysis of a large number of SEM micrographs. The size distribution of the aggregates is presented in Figure 3 at 5 vol% halloysite content. Size was characterized by the area occupied by the aggregates and it changes in a wide range. Homogeneity is much better in the melt mixed material than in the other two. The average size of the aggregates is smaller and more uniform than in the other two cases. Aggregates with size as large as 6000–8000 µm^2^ can be found in samples prepared by compression or film casting. Homogenization and sample preparation technology obviously has a huge effect on the homogeneity of the composites and, as a consequence, also on their properties.

The extent of aggregation depends also on filler content [40,41,42]. The effect is demonstrated in Table 1 (and Table S1). Average and most frequent aggregate size is listed in the table for the three technologies at two halloysite contents, at 5 and 10 vol%, respectively. The dimensions of about 100–150 aggregates were determined for each composition and technology. The larger efficiency of melt mixing in homogenization is also verified by the data in the table, which also confirm that the extent of aggregation increases with increasing filler content. Probably not very surprisingly, the most inhomogeneous materials are prepared by the grinding of the polymer, mixing it with the filler and compressing the mixture into films. Better homogeneity can be achieved by film casting, in which shear forces are quite small. Although melt mixing is quite advantageous, it cannot be used for all applications and polymers; for example, enzymes are quite sensitive to heat and might denature during processing. Nevertheless, the results strongly emphasize the need for the optimization of the processing technology used for the preparation of composites containing nanosized particles.

### 3.3. Thermal Analysis, Crystalline Structure

Besides homogeneity, the structure of the polymer also has a large impact on the performance of the material in any functional application. Crystallization modifies the distribution of the filler, as this latter is located in the amorphous phase. Crystallization and the exclusion of the filler from the crystalline phase can be especially important in electrospinning, in which the evaporation of the solvent and the simultaneous crystallization of the polymer determine the final structure of the composite. The glass transition temperature (T_g_) of PCL is low, below room temperature, which influences the release of any active component or the diffusion of reactants or metabolites during the degradation of the scaffold. Thermal analysis offers information on all these aspects of the polymer acting as the matrix of the composites.

**Figure 4:**
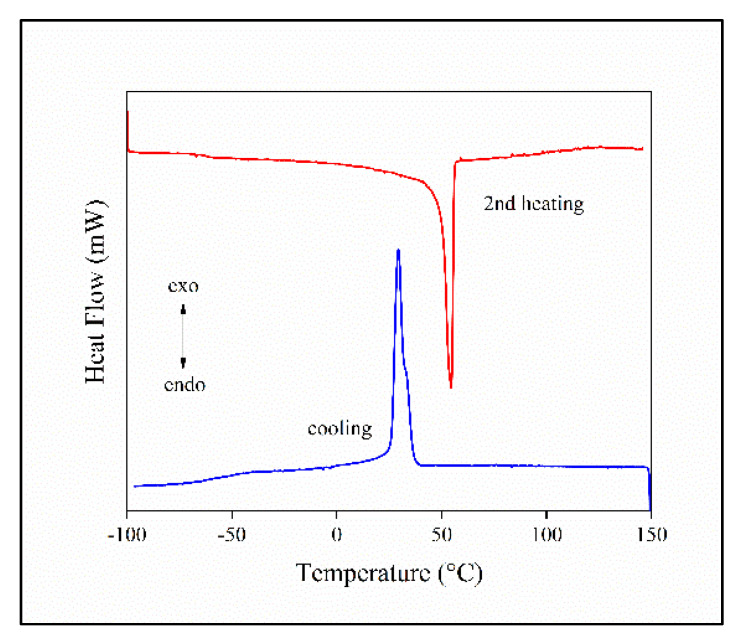
DSC melting and crystallization curves recorded on a PCL/halloysite composite prepared by film casting at 5 vol% halloysite content.

**Figure 5:**
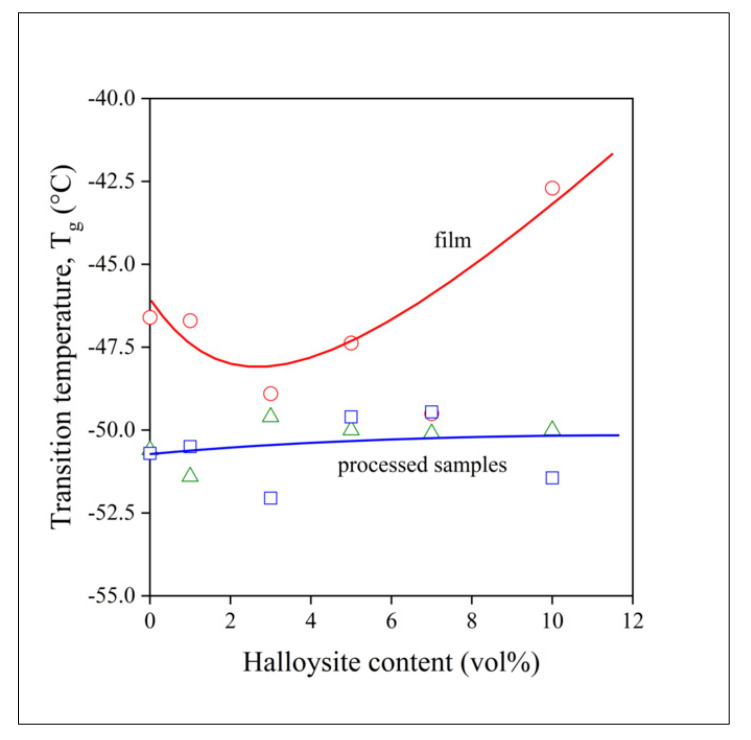
Glass transition temperature of PCL in PCL/halloysite composites determined by DMTA measurements from the maximum of loss tangent. Symbols: (○) film, (☐) compressed, (△) melt mixed.

The DSC curves of a composite containing 5 vol% halloysite and prepared by film casting is presented in Figure 4. The very fast crystallization and the melting of the polymer dominates the traces. Glass transition can be detected at below −50 °C, but its intensity is relatively small, because of the domination of the first order transitions in the figure. Nevertheless, glass transition can be detected and determined unambiguously (see Table S8 and Figures S28–S40). However, T_g_ was also determined by DMTA measurements, which are more accurate and convenient for the purpose (see Tables S9–S11 and Figures S41–S45). The T_g_ of the polymer is plotted against composition in Figure 5 for the three sets of materials prepared. Glass transition temperature is practically constant for the processed samples, i.e., melt mixed or compressed, but it changes somewhat with composition for the films. A slight minimum appears in the composition dependence of T_g_, which is quite difficult to understand. One would expect an increase in glass transition temperature with increasing filler content due to the adsorption of the polymer on the surface of the filler and a consequent decrease in molecular mobility. This latter effect accounts for the slight increase in T_g_ for the processed samples. However, a decrease in T_g_ was observed before [43], for example for PLA, and the same factors might result in the slight minimum here, also. Nevertheless, the T_g_ of the polymer is much below room temperature at all compositions, thus we might expect that diffusion and drug release will not be influenced much by the presence of the filler.

**Figure 6:**
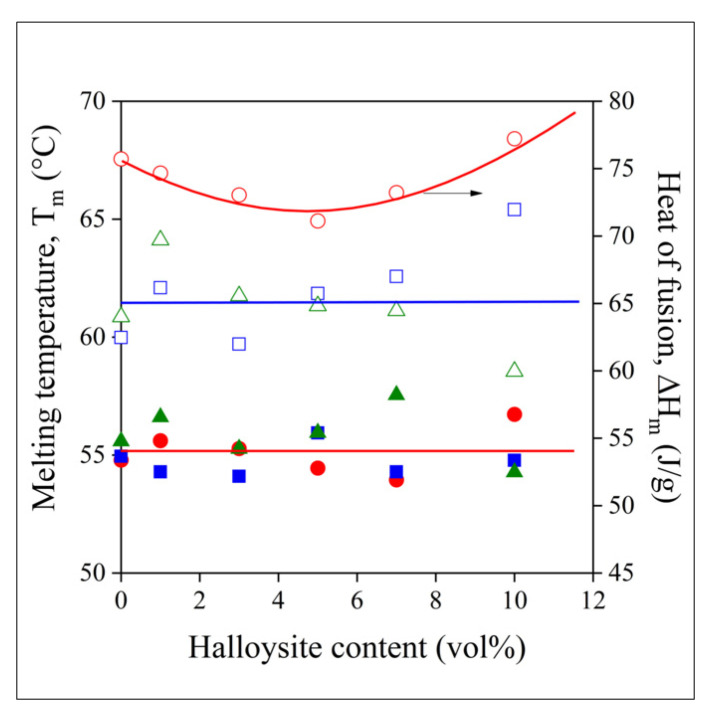
Melting characteristics of PCL halloysite composites plotted against halloysite content. Symbols: (○,●) film, (☐,■) compressed, (△,▲) melt mixed. Full symbols: heat of fusion; empty symbols: melting temperature.

The melting characteristics, the temperature (T_m_) and the heat of fusion (ΔH_m_) of the polymer are plotted against halloysite content in Figure 6. The characteristics were determined in the second heating run. Melting temperature barely changes with composition, and homogenization technology does not influence it either. The heat of fusion, on the other hand, differs for the samples prepared by melt technologies and film casting. The ΔH_m_ of the polymer in the films is larger, indicating larger crystallinity. Apparently, the presence of the solvent and its evaporation during film casting changes crystallization and crystalline structure, but also the mobility of the polymer molecules in the amorphous phase (see the composition dependence of T_g_, Figure 5). According to the results of thermal analysis, the structure and behavior of the polymer in samples prepared by solvent casting is different from those produced by melt processing, and we may expect the same for fibers prepared by electrospinning.

### 3.4. Tensile Properties

**Figure 7:**
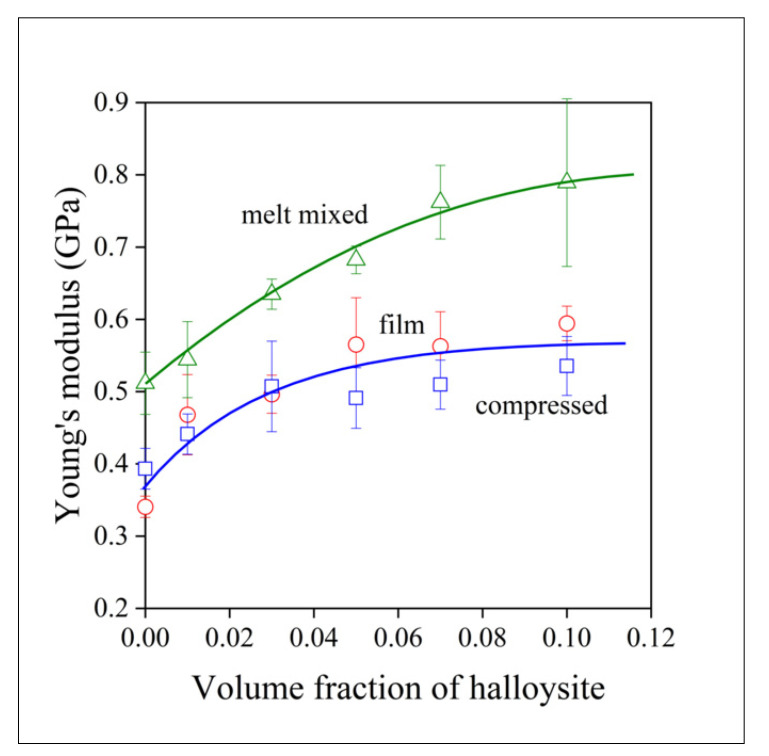
Effect of halloysite content on the stiffness of PCL/halloysite composites. Symbols: (○) film, (☐) compressed, (△) melt mixed.

The mechanical properties are important for composites used in structural applications, but often also when they are applied as functional materials. Moreover, mechanical properties may offer information about the structure of the material indirectly. The stiffness of the composites prepared is plotted against halloysite content in Figure 7. Interestingly, films and compressed samples have smaller Young’s modulus at the same filler content than melt mixed samples. Modulus mainly depends on composition, on the volume fraction of the filler in the polymer. However, with nanofillers, contact surface and interactions, as well as the structure of the polymer, also influence stiffness. Polymer structure cannot be the determining factor here, since films had larger crystallinity than samples prepared by the other two technologies. The differences in the extent of aggregation (see Table 1), however, may result in the dissimilar stiffness of the composites. The role of aggregation is confirmed by the composition dependence of stiffness as well. In the case of well-dispersed fillers, modulus should increase exponentially with increasing filler content [44,45]. The tendency towards saturation indicates increasing extent of aggregation with increasing halloysite content, in agreement with the results of Table 1; in addition, the change in slope is slower for melt mixed samples, indicating better homogeneity.

**Figure 8:**
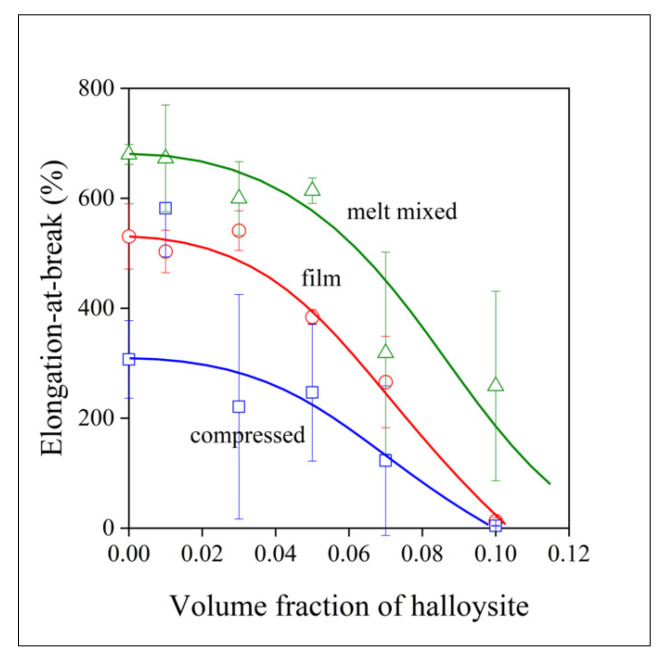
Dependence of the deformability of PCL/halloysite composites on filler content. Symbols: (○) film, (☐) compressed, (△) melt mixed.

Yield characteristics (yield stress and strain) as well as tensile strength and elongation-at-break convey the same message about the effect of homogenization technology and homogeneity as stiffness. Consequently, we refrain from presenting the composition dependence of all properties and show only that of elongation-at-break here, as it responds to heterogeneity more sensitively than the rest of the tensile properties (Figure 8, for further information see Tables S2–S4 and Figures S1–S14). The deformability of the samples prepared by the three technologies differ considerably from one another; the elongation-at-break of the melt mixed samples is twice as large as that of the compressed ones. Films are located in between the samples prepared by the other two technologies, thus offering a clear order in homogeneity agreeing well with the results of structural analysis (Table 1). The analysis of the composition dependence of tensile properties corroborates results obtained by other techniques and proves the superiority of melt mixing, and shows the largest extent of aggregation for the samples prepared by the direct compression of the mixture of the polymer and the halloysite powder. What remains is the checking of the influence of halloysite on electrospinning, as well as on the structure of the fibers and the mats produced by this technique.

### 3.5. Fiber Spinning

**Figure 9:**
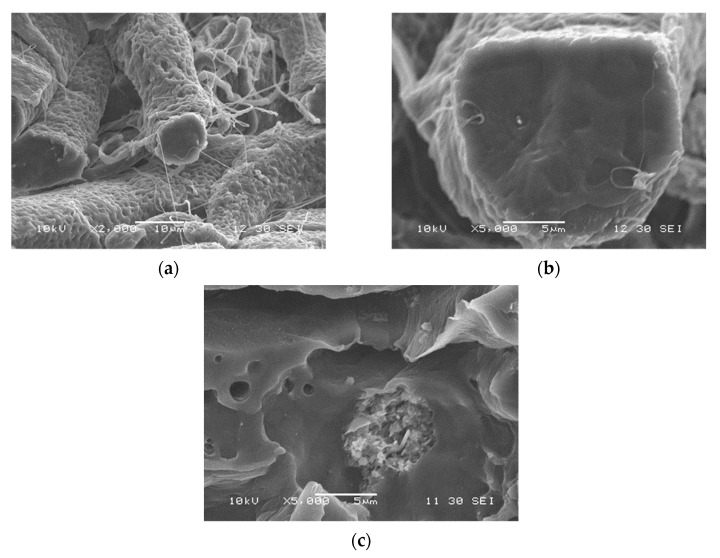
Effect of electrospinning on the distribution of halloysite nanotubes and the structure of the fibers prepared from PCL/halloysite composite materials. Halloysite content and magnification: (**a**) 3 vol%, 2000×; (**b**) 3 vol% 5000×; (**c**) 5 vol%, 5000×.

**Figure 10:**
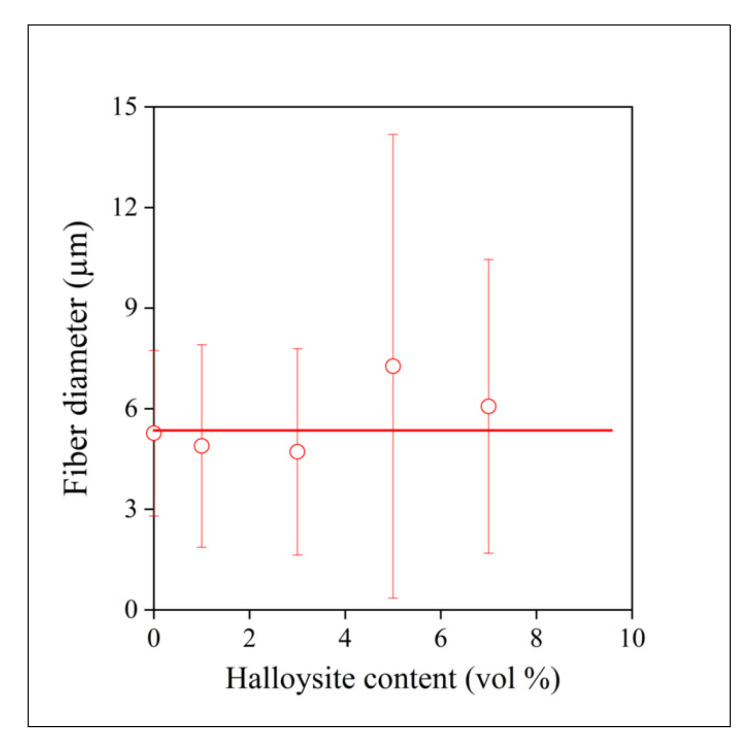
The composition dependence of fiber diameter of electrospun fibers.

A solution was prepared from the polymer, and halloysite tubes were added to it to prepare a suspension. Fibers could be spun from the mixture with a more or less regular morphology. As photos of electrospun fibers have been published in large numbers in the literature before, we focus more on homogeneity, the distribution of the filler in the fibers, and on aggregation. SEM micrographs recorded on the fractured surface of fiber mats at various magnifications and halloysite contents are presented in Figure 9. The mats were slightly compressed, cooled down to liquid nitrogen temperature, and fractured in order to see the cross-section of the fibers and the dispersion of the filler in them. The fibers seen in Figure 9a have irregular surfaces containing a large number of dips or holes that are the consequence of the evaporation of the solvent and the crystallization of the polymer. Smaller fiber-like structural entities are located among the larger fibers, which can be excluded filler particles or thinner polymer fibers, but their identification needs further study. The cross section of a broken fiber is shown in Figure 9b. The surface is smooth and seems to be void of filler particles, supporting the assumption that a part of the filler is not located inside, but among the fibers. Figure 9c presents another broken fiber containing aggregated filler particles showing that homogenization is an issue even in the solution preparation and fiber spinning of PCL/halloysite composites. The presence of such aggregates might considerably influence the properties and performance of the composites. Fiber diameter and its distribution were also determined in order to characterize the spun fibers even further. The composition dependence of average fiber diameter is presented in Figure 10. Halloysite content does not change the diameter of the fibers practically at all, thus the main factors determining fiber properties are structure and aggregation.

**Figure 11:**
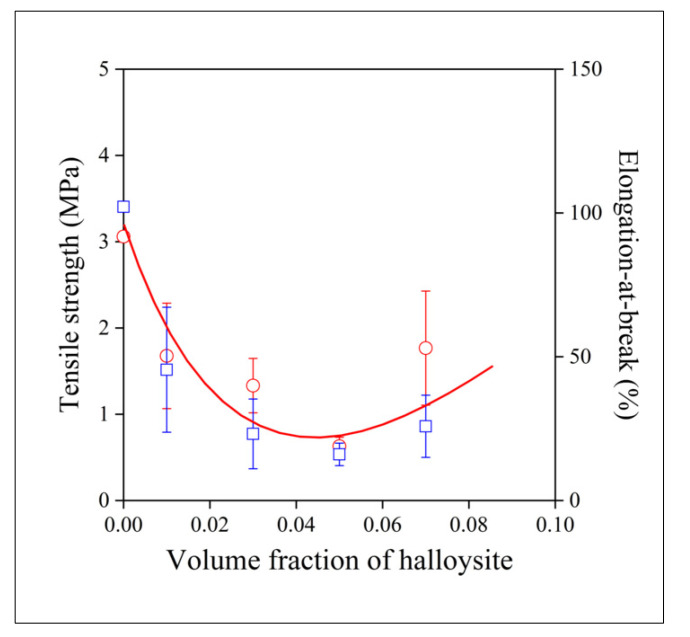
Effect of filler content on the mechanical properties of fiber mats prepared from electrospun PCL/halloysite fibers. Symbols: (○) tensile strength, (☐) elongation-at-break.

An attempt was made to estimate this effect quantitatively. Fiber mats with approximately the same weight were compressed into rectangular samples and their mechanical characteristics were determined by tensile testing. The strength and the elongation of the samples are presented in Figure 11 as a function of halloysite content. Only one line is drawn in the figure to guide the eye and to avoid confusion, but the correlation is very similar for the two properties. Fibers could not be spun from dispersions containing more than 7 vol% filler. All numbers, especially strength, are only indicative values, since neither the exact number nor the orientation of the fibers in the mats are known. Strength is small and decreases considerably with increasing halloysite content. The decrease might be the result of the formation of aggregates (see Figure 9c), but the filler may also influence the fiber spinning process, the location of the filler, evaporation, and the crystallization of the polymer. As with strength, elongation-at-break, which is very sensitive to homogeneity, also decreases drastically with increasing filler content. The larger values of strength and deformability measured at the largest halloysite content might be the net effect of the competing factors noted above. Halloysite content and spinning conditions must be carefully optimized if such composites are to be used for the preparation of resorbable scaffolds, and homogeneity is a crucial question in accordance with the results presented above.

## 4. Discussion

The study of the structure of PCL/halloysite nanotube composites prepared by a number of homogenization technologies showed that various number of aggregates form in them depending on the technology and on halloysite content. The size and number of aggregates increase with increasing halloysite content. Melt mixing results in more homogeneous composites than the simple compression of the component powders or homogenization in solution and film casting. Homogeneity and the extent of aggregation determines all properties, including functionality. The mechanical properties of the polymer deteriorate with increasing extent of aggregation; even stiffness depends on homogeneity, whereas strength and deformability decrease drastically as the number and the size of the aggregates increase. Aggregation decreases the contact surface between the polymer and the filler, while large aggregates act as defects and initiate failure during loading. Not only dispersed structure, but also the physical state and crystalline structure of the polymer also influence homogeneity and properties. The presence of the filler affects the preparation of electrospun fibers for scaffolds. A part of the filler is excluded from the fibers while another part forms aggregates that complicates fiber spinning and deteriorates properties. The results indicate that spinning is easier and the quality of the fibers is better if a material homogenized previously by melt mixing is used for the production of the fibers.

## Figures and Tables

**Table 1:** Effect of halloysite content and sample preparation technology on aggregation in PCL/halloysite nanocomposites.

Technology	Halloysite (vol%)	Aggregate Area (μm)	
		Average	Most Frequent
Film casting	5	499.9 ± 652.3	88.5
Compression		391.8 ± 687.1	98.1
Melt mixing		83.2 ± 117.8	9.6
Film casting	10	547.9 ± 874.1	162.4
Compression		1078.1 ± 2348.4	443.3
Melt mixing		322.2 ± 398.4	137.3

## Data Availability

The data presented in this study are available on request from the corresponding author. The data are not publicly available due to legal reasons.

## Supplementary Materials

The following are available online at https://www.mdpi.com/article/10.3390/polym13213772/s1, Figure S1: Stress vs. strain curves of the PCL/halloysite composites with different filler content prepared by film casting; Figure S2: Stress vs. strain curves of the PCL/halloysite composites with different filler content prepared by compression molding; Figure S3: Stress vs. strain curves of the PCL/halloysite composites with different filler content prepared by internal mixer; Figure S4: Stress vs. strain curves of the PCL/halloysite composites prepared by different tech-nique at 0 vol% filler content; Figure S5: Stress vs. strain curves of the PCL/halloysite composites prepared by different tech-nique at 1 vol% filler content; Figure S6: Stress vs. strain curves of the PCL/halloysite composites prepared by different tech-nique at 3 vol% filler content; Figure S7: Stress vs. strain curves of the PCL/halloysite composites prepared by different tech-nique at 5 vol% filler content; Figure S8: Stress vs. strain curves of the PCL/halloysite composites prepared by different tech-nique at 7 vol% filler content; Figure S9: Stress vs. strain curves of the PCL/halloysite composites prepared by different tech-nique at 10 vol% filler content; Figure S10: Effect of halloysite content on the Young’s modulus of PCL/halloysite composites. Symbols:(■) film casting, (▲) internal mixer, (●) compression molding; Figure S11: Effect of halloysite content on yield stress of PCL/halloysite composites. Symbols:(■) film casting, (▲) internal mixer, (●) compression molding; Figure S12: Effect of halloysite content on yield strain of PCL/halloysite composites. Symbols:(■) film casting, (▲) internal mixer, (●) compression molding; Figure S13: Effect of halloysite content on tensile strength of PCL/halloysite composites. Sym-bols:(■) film casting, (▲) internal mixer, (●) compression molding; Figure S14: Effect of halloysite content on tensile strain of PCL/halloysite composites. Symbols:(■) film casting, (▲) internal mixer, (●) compression molding; Figure S15: The principle of the determination of onset value. PCL/halloysite composite at 1 vol% halloysite content prepared by compression molding; Figure S16: Onset temperature values of PCL/halloysite composites. Symbols:(■) film casting, (▲) internal mixer, (●) compression molding; Figure S17: Onset weight values of PCL/halloysite composites. Symbols:(■) film casting, (▲) in-ternal mixer, (●) compression molding; Figure S18: The residual material mass of PCL/halloysite composites as function of filler content. Symbols:(■) film casting, (▲) internal mixer, (●) compression molding; Figure S19: TGA curves of the PCL/halloysite composites with different filler content prepared by compression molding; Figure S20: TGA curves of the PCL/halloysite composites with different filler content prepared by film casting; Figure S21: TGA curves of the PCL/halloysite composites with different filler content prepared by internal mixer; Figure S22: Characteristic TGA curves of the PCL/halloysite composites prepared by different technique at 0 vol% filler content; Figure S23: Characteristic TGA curves of the PCL/halloysite composites prepared by different technique at 1 vol% filler content; Figure S24: Characteristic TGA curves of the PCL/halloysite composites prepared by different technique at 3 vol% filler content; Figure S25: Characteristic TGA curves of the PCL/halloysite composites prepared by different technique at 5 vol% filler content; Figure S26: Characteristic TGA curves of the PCL/halloysite composites prepared by different technique at 7 vol% filler content; Figure S27: Characteristic TGA curves of the PCL/halloysite composites prepared by different technique at 10 vol% filler content; Figure S28: Effect of halloysite content on crystallization temperature of PCL/halloysite composite. Symbols:(■) film casting, (▲) internal mixer, (●) compression molding; Figure S29: Effect of halloysite content on crystallization enthalpy (ΔHc) of PCL/halloysite compo-site. Symbols:(■) film casting, (▲) internal mixer, (●) compression molding; Figure S30: Effect of halloysite content on transition temperature of PCL/halloysite composite de-rived from second heating. Symbols:(■) film casting, (▲) internal mixer, (●) compression molding; Figure S31: Effect of halloysite content on melting temperature of PCL/halloysite composite de-rived from second heating. Symbols:(■) film casting, (▲) internal mixer, (●) compression molding; Figure S32: Effect of halloysite content on melting enthalpy (ΔHm) of PCL/halloysite composite de-rived from second heating. Symbols:(■) film casting, (▲) internal mixer, (●) compression molding; Figure S33: Crystallization curves of PCL/Halloysite composites with different filler content pre-pared by compression molding; Figure S34: Crystallization curves of PCL/Halloysite composites with different filler content pre-pared by film casting; Figure S35: Crystallization curves of PCL/Halloysite composites with different filler content pre-pared by internal mixer; Figure S36: Second heating run of DSC curves of PCL/Halloysite composites with different filler content prepared by compression molding; Figure S37: Second heating run of DSC curves of PCL/Halloysite composites with different filler content prepared by film casting; Figure S38: Second heating run of DSC curves of PCL/Halloysite composites with different filler content prepared by internal mixer; Figure S39: Crystallization temperature (Tc) and crystallization enthalpy (ΔHc) of PCL/Halloysite composites as a function of halloysite content. Symbols: Tc: (■) film casting, (▲) internal mixer, (●) compression molding, ΔHc: (□) film casting, (Δ) internal mixer, (○) compression molding; Figure S40: Melting temperature (Tm) and melting enthalpy (ΔHm) of PCL/Halloysite composites as a function of halloysite content. Symbols: Tc: (■) film casting, (▲) internal mixer, (●) compres-sion molding, ΔHc: (□) film casting, (Δ) internal mixer, (○) compression molding; Figure S41: Results of DMA measurements of PCL/halloysite composites prepared by film casting. (a) tan δ, (b) loss modulus; Figure S42: Results of DMA measurements of PCL/halloysite composites prepared by compression molding. (a) tan δ, (b) loss modulus; Figure S43: Results of DMA measurements of PCL/halloysite composites prepared by internal mixer. (a) tan δ, (b) loss modulus; Figure S44: Glass transition temperatures (Tg) derived from tan δ values of PCL/halloysite compo-sites. Symbols: (■) film casting, (▲) internal mixer, (●) compression molding; Figure S45: Glass transition temperatures (Tg) derived from E’’ values of PCL/halloysite compo-sites. Symbols: (■) film casting, (▲) internal mixer, (●) compression molding; Table S1: Effect of halloysite content and sample preparation technology on aggregation in PCL/halloysite nanocomposites; Table S2: Results of tensile test of PCL/halloysite composites prepared by film casting; Table S3: Results of tensile test of PCL/halloysite composites prepared by compression molding; Table S4: Results of tensile test of PCL/halloysite composites prepared by internal mixer; Table S5: Results of TGA measurements of PCL/halloysite composites prepared by film casting; Table S6: Results of TGA measurements of PCL/halloysite composites prepared by compression molding; Table S7: Results of TGA measurements of PCL/halloysite composites prepared by internal mixer; Table S8: Results of DSC measurements of PCL/halloysite composites with variant filler content prepared by different technology; Table S9: Results of DMA measurements of PCL/halloysite composites prepared by film casting; Table S10: Results of DMA measurements of PCL/halloysite composites prepared by compression molding; Table S11: Results of DMA measurements of PCL/halloysite composites prepared by internal mix-er.

## References

[1] George A., Sanjay M.R., Srisuk R., Parameswaranpillai J., Siengchin S. (2020). A comprehensive review on chemical properties and applications of biopolymers and their composites. Int. J. Biol. Macromol..

[2] Niaounakis M. (2015). Biopolymers: Applications and Trends.

[3] Elvers D., Song C.H., Steinbüchel A., Leker J. (2016). Technology trends in biodegradable polymers: Evidence from patent analysis. Polym. Rev..

[4] Udayakumar G.P., Muthusamy S., Selvaganesh B., Sivarajasekar N., Rambabu K., Banat F., Sivamani S., Sivakumar N., Hosseini-Bandegharaei A., Show P.L. (2021). Bipolymers and composites: Properties, characterization and their applications in food, medical and pharmaceutical industries. J. Environ. Chem. Eng..

[5] Park S.B., Lih E., Park K.S., Joung Y.K., Han D.K. (2017). Biopolymer-based functional composites for medical applications. Prog. Polym. Sci..

[6] Sodhi A.S., Sharma N., Bhatia S., Verma A., Soni S., Batra N. (2021). Insights on sustainable approaches for production and applications of value added products. Chemosphere.

[7] Tan H.C., Ananthakrishna R. (2017). A review of bioresorbable scaffolds: Hype or hope?. Singap. Med. J..

[8] Narayanan G., Vernekar V.N., Kuyinu E.L., Laurencin C.T. (2016). Poly(lactic-acid)-based biomaterials for orthopaedic regenerative engineering. Adv. Drug Deliv. Rev..

[9] Ovsianikov A., Khademhosseini A., Mironov V. (2018). The synergy of scaffold-based and scaffold-free tissue engineering strategies. Trends Biotechnol..

[10] Woodruff M.A., Hutmatcher D.W. (2010). The return of a forgotten polymer- Polycaprolactone in the 21st century. Prog. Polym. Sci..

[11] Manoukian O.S., Sardashti N., Stedman T., Gailiunas K., Ojha A., Penalosa A., Mancuso C., Hobert M., Kumbar S.G. (2019). Biomaterials for Tissue Engineering and Regenerative Medicine. Ency. Biom. Eng..

[12] Gunatillake P.A., Adhikari R. (2003). Biodegradable synthetic polymers for tissue engineering. Eur. Cell Mater..

[13] Pastorino L., Pioli F., Zilli M., Converti A., Nicolini C. (2004). Lipase-catalyzed degradation of poly(ε-caprolactione). Enzyme Microb. Technol..

[14] Aris M.H., Annuar M.S.M., Ling T.C. (2016). Lipase-mediated degradation of poly-ε-caprolactone in toluene: Behavior and its action mechanism. Polym. Degrad. Stab..

[15] Hegyesi N., Hodosi E., Polyák P., Faludi G., Balogh-Weiser D., Pukánszky B. (2020). Controlled degradation of poly-ε-caprolactone for resorbable scaffolds. Colloids Surf. B Biointerfaces.

[16] Liu M., Duan X.-P., Li Y.-M., Yang D.-P., Long Y.-Z. (2017). Electrospun nanofibers for wound healing. Mater. Sci. Eng. C.

[17] Zahedi P., Rezaeian I., Ranaei-Siadat S.O., Jafari S.H., Supaphol P. (2010). A review on wound dressings with an emphasis on electrospun nanofibrous polymeric bandages. Polym. Adv. Technol..

[18] Thakkar S., Misra M. (2017). Electrospun polymeric nanofibers: New horizons in drug delivery. Eur. J. Pharm. Sci..

[19] Sill T.J., von Recum H.A. (2008). Electrospinning: Applications in drug delivery and tissue engineering. Biomaterials.

[20] Zamani M., Prabhakaran M.P., Ramakrishna S. (2013). Advances in drug delivery via electrospun and electrosprayed nanomaterials. Int. J. Nanomed..

[21] Amatulu R., Khan W.S. (2019). Introduction to electrospun nanofibers. Synthesis and Applications of Electrospun Nanofibers.

[22] Chou S.F., Carson D., Woodrow K.A. (2015). Current strategies for sustaining drug release from electrospun nanofibers. J. Control. Release.

[23] Chou S.F., Woodrow K.A. (2017). Relationships between mechanical properties and drug release from electrospun fibers of PCL and PLGA blends. J. Mech. Behav. Biomed. Mater..

[24] Zhai R., Zhang B., Liu L., Xie Y., Zhang H., Liu J. (2010). Immobilization of enzyme biocatalyst on natural halloysite nanotubes. Catal. Commun..

[25] Chao C., Liu J., Wang J., Zhang Y., Zhang B., Zhang Y., Xiang X., Chen R. (2013). Surface modification of halloysite nanotubes with dopamine for enzyme immobilization. ACS Appl. Mater. Interfaces.

[26] Tully J., Yendluri R., Lvov Y. (2016). Halloysite clay nanotubes for enzyme immobilization. Biomacromolecules.

[27] Machado G.S., de Freitas Castro K.A.D., Wypych F., Nakagaki S. (2008). Immobilization of metalloporphyrins into nanotubes of natural halloysite toward selective catalysts for oxidation reactions. J. Mol. Catal. A Chem..

[28] Santos A.C., Ferreira C., Veiga F., Ribeiro A.J., Panchal A., Lvov Y., Agarwal A. (2018). Halloysite clay nanotubes for life sciences applications: From drug encapsulation to bioscaffold. Adv. Colloid Interface Sci..

[29] Colijn I., Schroën K. (2021). Thermoplastic bio-nanocomposites: From measurement of fundamental properties to practical application. Adv. Colloid Interface Sci..

[30] Shrestha S., Wang B., Dutta P. (2020). Nanoparticle processing: Understanding and controlling aggregation. Adv. Colloid Interface Sci..

[31] Liu M., Jia Z., Jia D., Zhou C. (2014). Recent advance in research on halloysite nanotubes-polymer nanocomposite. Prog. Polym. Sci..

[32] Iyer S., Schiraldi D.A. (2007). Role of specific interactions and solubility in the reinforcement of bisphenol A polymers with polyhedral oligomeric silsesquioxanes. Macromolecules.

[33] Schmidt R.G., Gordon G.V., Dreiss C.A., Cosgrove T., Krukonis V.J., Williams K., Wetmore P.M. (2010). A critical size ratio for viscosity reduction in poly(dimethylsiloxane)-polysilicate nanocomposites. Macromolecules.

[34] Sun H., Jiao R., An G., Xu H., Wang D. (2021). Influence of particle size on the aggregation behavior of nanoparticles: Role of structural hydration layer. J. Environ. Sci..

[35] Krzysko A.J., Nakouzi E., Zhang X., Graham T.R., Rosso K.M., Schenter G.K., Ilavsky J., Kuzmenko I., Frith M.G., Ivory C.F. (2020). Correlating inter-particle forces and particle shape to shear-induced aggregation/fragmentation and rheology for dilute anisotropic particle suspensions: A complementary study via capillary rheometry and *in-situ* small and ultra-small angle X-ray scattering. J. Colloid Interface Sci..

[36] Usune S., Kubo M., Tsukada T., Koike O., Tatsumi R., Fujita M., Takami S., Adschiri T. (2019). Numerical simulations of dispersion and aggregation behavior of surface-modified nanoparticles under shear flow. Powder Technol..

[37] Yang Y.T., Cheng Y., Len F., Huang L., Wang Z.J., Tian W.Q. (2017). Recent advances on modification of halloysite nanotubes for multifunctional applications. Appl. Sci..

[38] Lvov Y., Guo B., Fakhrullin R.F. (2017). Functional Polymer Composites with Nanoclays.

[39] Tharmavaram M., Pandey G., Rawtani D. (2018). Surface modified halloysite nanotubes: A flexible interface for biological, environmental and catalytic applications. Adv. Colloid Interface Sci..

[40] Móczó J., Fekete E., László K., Pukánszky B. (2003). Aggregation of particulate fillers: Factors, determination, properties. Macromol. Symp..

[41] Shandiz S.A., Moradi M.A., Babaluo A.A., Jalili A.H. (2015). A comparative experimental and molecular simulation study on the mechanical and morphological behaviors of adamantane-based polypropylene composites. Comput. Mater. Sci..

[42] Zhang S., Cao X.Y., Ma Y.M., Ke Y.C., Zhang J.K., Wang F.S. (2011). The effects of particle size and content on the thermal conductivity and mechanical properties of Al_2_O_3_/high density polyethylene (HDPE) composites. Express Polym. Lett..

[43] Müller P., Imre B., Bere J., Móczó J., Pukánszky B. (2015). Physical ageing and molecular mobility in PLA blends and composites. J. Therm. Anal. Calorim..

[44] Pukánszky B., Fekete E. (1998). Aggregation tendency of particulate fillers: Determination and consequences. Period. Polytech. Chem. Eng..

[45] Nielsen L.E. (1974). Mechanical Properties of Polymers and Composites.

