# Supplementary material for: Poly-ε-Caprolactone/Halloysite Nanotube Composites for Resorbable Scaffolds: Effect of Processing Technology on Homogeneity and Electrospinning
Source: Polymers (Basel). 2021 Oct 31;13(21):3772. doi: 10.3390/polym13213772 (PMC8587687; doi:10.3390/polym13213772)
Supplement: Supplementary file 1 [file polymers-13-03772-s001.zip › polymers-1400552-supplementary.pdf]

Supplementary Information

Poly-ε-Caprolactone/Halloysite Nanotube Composites for Resorbable Scaffolds: Effect of Processing Technology on Homogeneity and Electrospinning

Muriel Józó [1,2], Róbert Várdai [1,2], Nóra Hegyesi [1,2], János Móczó [1,2] and Béla Pukánszky [1,2,*]

[1] Laboratory of Plastics and Rubber Technology, Department of Physical Chemistry and Materials Science, Budapest University of Technology and Economics, P.O. Box 91, H-1521 Budapest, Hungary; jozo.muriel@vbk.bme.hu (M.J.); vardai.robert@vbk.bme.hu (R.V.); hegyesi.nora@vbk.bme.hu (N.H.); jmoczo@edu.bme.hu (J.M.)
[2] Institute of Materials and Environmental Chemistry, Research Centre for Natural Sciences, ELKH Eötvös Loránd Research Network, P.O. Box 286, H-1519 Budapest, Hungary
* Correspondence: pukanszky.bela@vbk.bme.hu; Tel.: +36-(14)-632015

Citation: Józó, M.; Várdai, R.; Hegyesi, N.; Móczó, J.; Pukánszky, B. Poly-ε-Caprolactone/Halloysite Nanotube Composites for Resorbable Scaffolds: Effect of Processing Technology on Homogeneity and Electrospinning. *Polymers* **2021**, *13*, 3772. https://doi.org/10.3390/polym13213772

Academic Editors: Laura Aliotta; Vito Gigante and Andrea Lazzeri

Received: 14 September 2021
Accepted: 28 October 2021
Published: 31 October 2021

Publisher's Note: MDPI stays neutral with regard to jurisdictional claims in published maps and institutional affiliations.

Copyright: © 2021 by the authors. Licensee MDPI, Basel, Switzerland. This article is an open access article distributed under the terms and conditions of the Creative Commons Attribution (CC BY) license (http://creativecommons.org/licenses/by/4.0/).

1. The extent of aggregation

Table S1. Effect of halloysite content and sample preparation technology on aggregation in PCL/halloysite nanocomposites.

Technology	Halloysite (vol%)	Aggregate area (μm²) Average	Most frequent
Film casting	1	442.6 ± 577.9	58.8
Compression		308.2 ± 497.5	62.1
Melt mixing		54.7 ± 76.7	5.8
Film casting	3	471.7 ± 586.3	72.9
Compression		350.3 ± 513.5	77.3
Melt mixing		72.0 ± 89.8	7.2
Film casting	5	499.9 ± 652.3	88.5
Compression		391.8 ± 687.1	98.1
Melt mixing		83.2 ± 117.8	9.6
Film casting	7	521.1 ± 789.0	141.0
Compression		769.6 ± 1411.2	278.9
Melt mixing		215.0 ± 364.4	46.4
Film casting	10	547.9 ± 874.1	162.4
Compression		1078.1 ± 2348.4	443.3
Melt mixing		322.2 ± 398.4	137.3

2. Results of tensile tests

Table S2. Results of tensile test of PCL/halloysite composites prepared by film casting.

**Halloysite content**		**Modulus, E (GPa)**		**Yield stress, σ_y (MPa)**		**Yield strain, ε_y (%)**		**Tensile strength, σ (MPa)**		**Strain, ε (%)**	
**Film casting**	**(v%)**	**Avg.**	**Dev.**	**Avg.**	**Dev.**	**Avg.**	**Dev.**	**Avg.**	**Dev.**	**Avg.**	**Dev.**
0 HAL	0.00	0.34	0.01	11.25	0.70	9.70	2.35	26.67	3.48	530.66	59.41
1 HAL	1.00	0.47	0.06	13.37	0.80	7.95	0.93	25.87	3.17	503.30	38.65
3 HAL	3.00	0.50	0.03	14.09	0.30	9.20	1.17	26.85	2.05	541.19	36.00
5 HAL	5.00	0.57	0.06	13.92	0.49	5.36	0.39	19.46	0.56	384.20	13.70
7 HAL	7.00	0.56	0.05	12.64	1.53	5.45	0.78	14.23	3.03	265.78	83.11
10 HAL	10.00	0.59	0.02	11.54	0.79	4.56	1.28	10.78	0.74	13.01	5.83

Table S3. Results of tensile test of PCL/halloysite composites prepared by compression molding.

**Halloysite content**		**Modulus, E (GPa)**		**Yield stress, σ_y (MPa)**		**Yield strain, ε_y (%)**		**Tensile strength, σ (MPa)**		**Strain, ε (%)**	
**Compression molding**	**(v%)**	**Avg.**	**Dev.**	**Avg.**	**Dev.**	**Avg.**	**Dev.**	**Avg.**	**Dev.**	**Avg.**	**Dev.**
0 HAL	0.00	0.39	0.03	14.62	0.77	10.02	0.64	17.80	4.56	306.95	70.53
1 HAL	1.00	0.44	0.03	13.45	0.68	8.02	1.48	33.68	5.25	581.85	88.31
3 HAL	3.00	0.51	0.06	13.03	1.22	6.50	0.75	19.21	5.17	220.85	204.18
5 HAL	5.00	0.49	0.04	12.68	0.44	6.12	1.07	14.95	3.28	246.83	124.82
7 HAL	7.00	0.51	0.03	11.35	1.30	4.69	1.56	11.98	1.24	122.86	136.13
10 HAL	10.00	0.54	0.04	11.35	0.69	3.50	0.36	11.67	0.95	4.29	0.82

Table S4. Results of tensile test of PCL/halloysite composites prepared by internal mixer.

**Halloysite content**		**Modulus, E (GPa)**		**Yield stress, σ_y (MPa)**		**Yield strain, ε_y (%)**		**Tensile strength, σ (MPa)**		**Strain, ε (%)**	
**Internal mixer**	**(v%)**	**Avg.**	**Dev.**	**Avg.**	**Dev.**	**Avg.**	**Dev.**	**Avg.**	**Dev.**	**Avg.**	**Dev.**
0 HAL	0.00	0.51	0.04	16.12	0.54	10.27	1.16	42.19	1.25	679.86	18.04
1 HAL	1.00	0.54	0.05	15.89	0.63	10.06	0.39	39.19	5.89	672.66	97.21
3 HAL	3.00	0.64	0.02	16.98	0.12	8.90	0.38	35.42	4.55	599.66	66.96
5 HAL	5.00	0.68	0.02	16.37	0.60	8.54	0.28	34.96	2.26	613.75	23.07
7 HAL	7.00	0.76	0.05	17.13	1.40	6.73	1.42	21.14	3.74	318.57	183.47
10 HAL	10.00	0.79	0.12	16.24	1.27	6.10	1.29	18.68	3.39	258.53	172.33

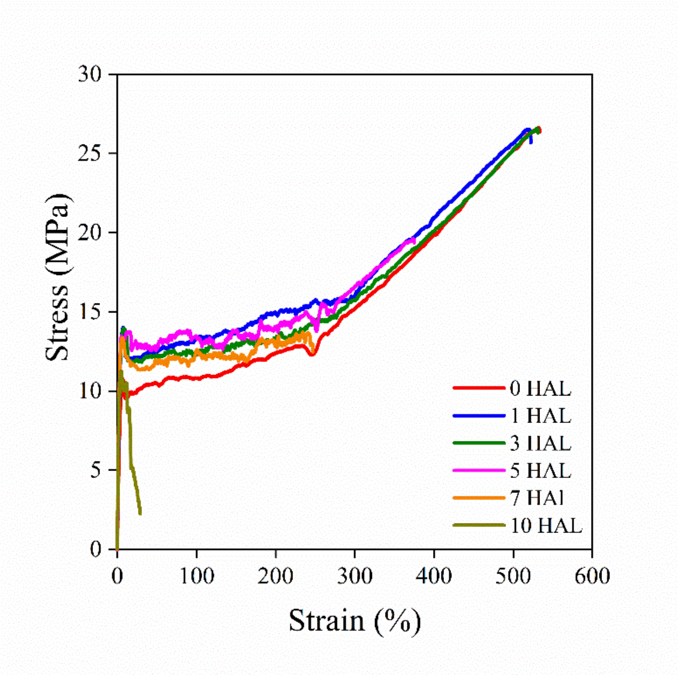

Figure S1. Stress vs. strain curves of the PCL/halloysite composites with different filler content prepared by film casting.

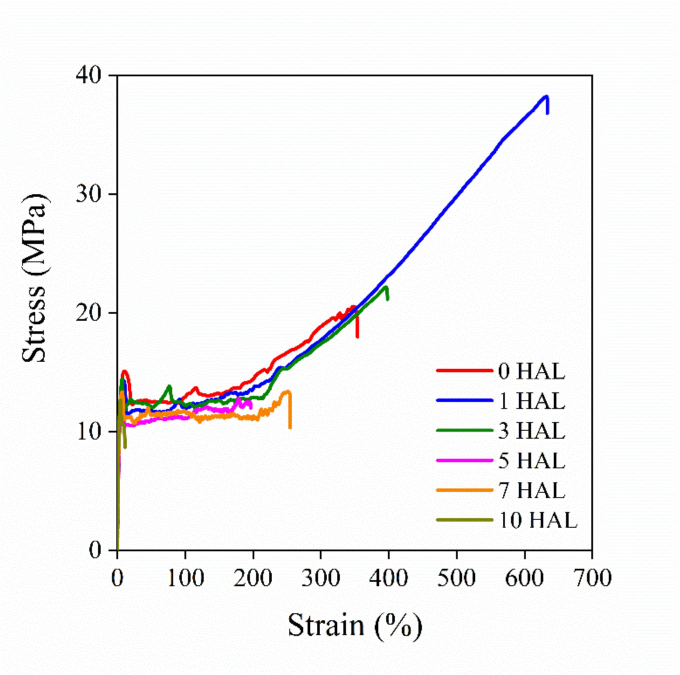

Figure S2. Stress vs. strain curves of the PCL/halloysite composites with different filler content prepared by compression molding.

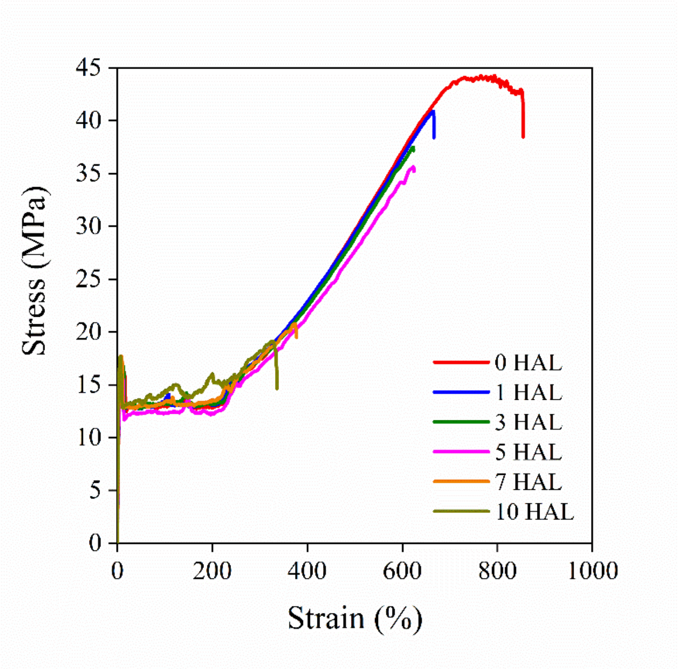

Figure S3. Stress vs. strain curves of the PCL/halloysite composites with different filler content prepared by internal mixer.

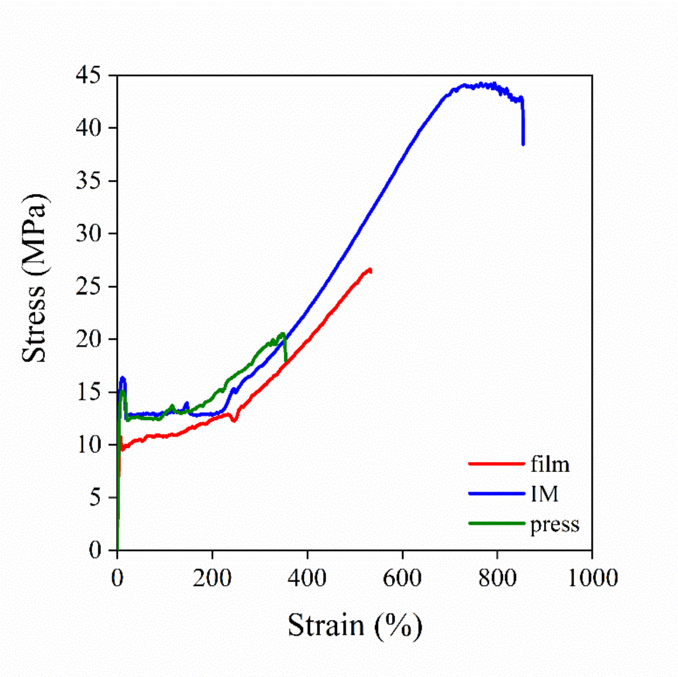

Figure S4. Stress vs. strain curves of the PCL/halloysite composites prepared by different technique at 0 vol% filler content.

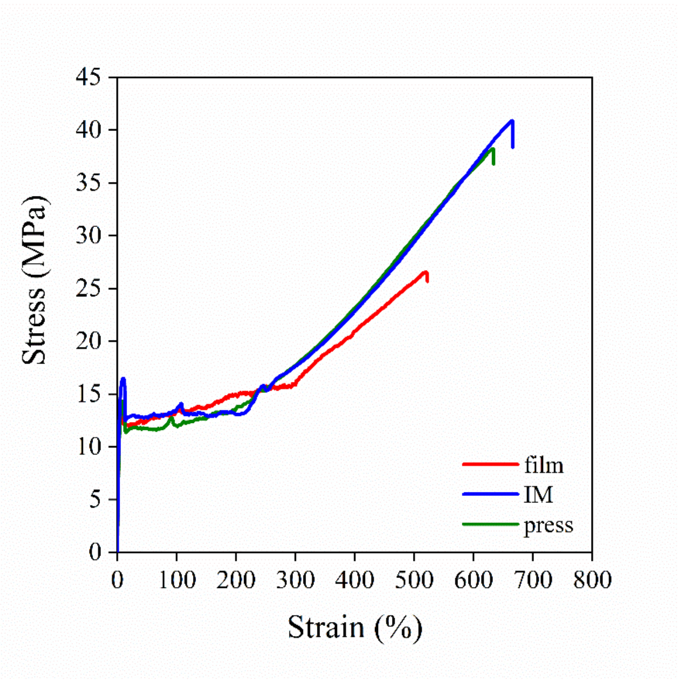

Figure S5. Stress vs. strain curves of the PCL/halloysite composites prepared by different technique at 1 vol% filler content.

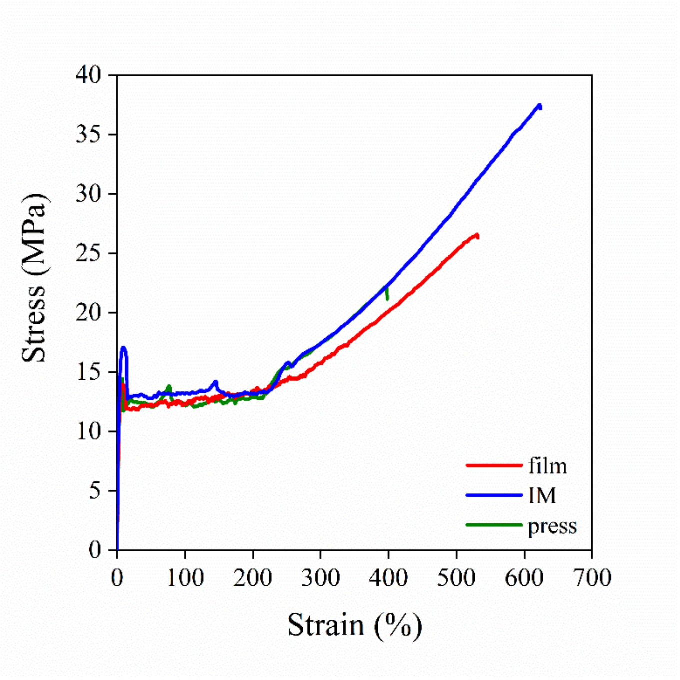

Figure S6. Stress vs. strain curves of the PCL/halloysite composites prepared by different technique at 3 vol% filler content.

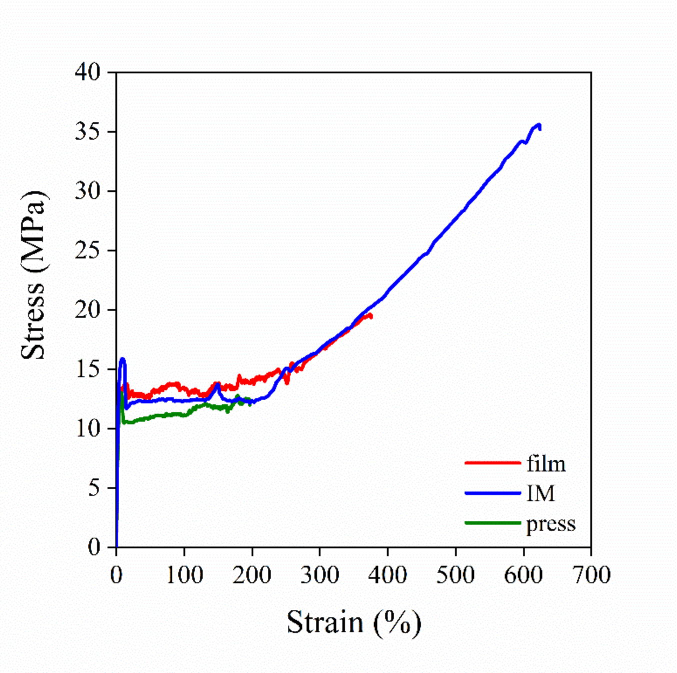

Figure S7. Stress vs. strain curves of the PCL/halloysite composites prepared by different technique at 5 vol% filler content.

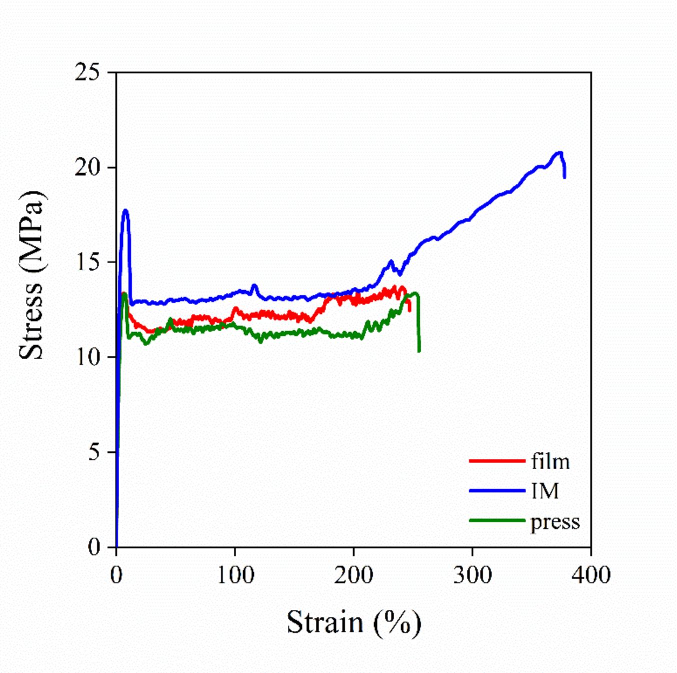

Figure S8. Stress vs. strain curves of the PCL/halloysite composites prepared by different technique at 7 vol% filler content.

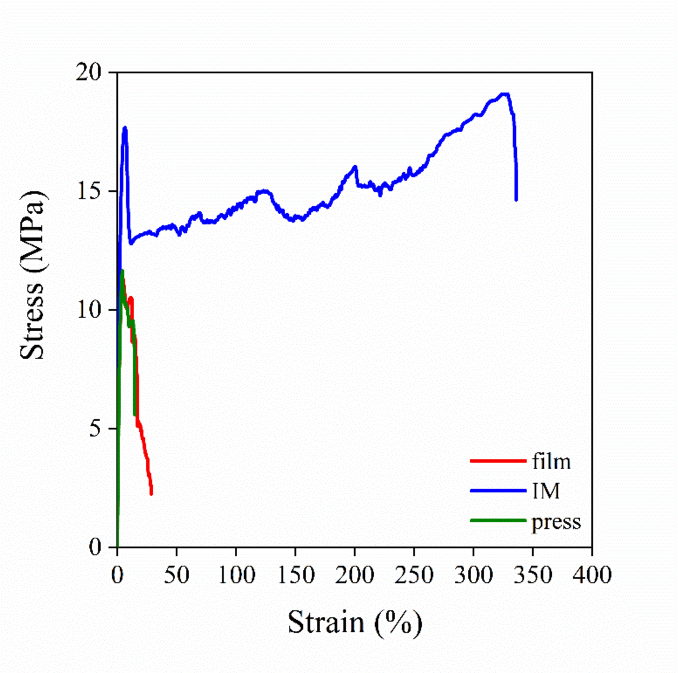

Figure S9. Stress vs. strain curves of the PCL/halloysite composites prepared by different technique at 10 vol% filler content.

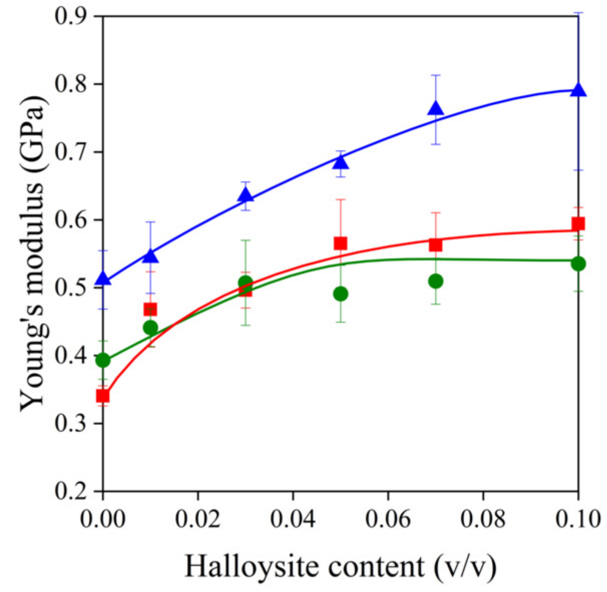

Figure S10. Effect of halloysite content on the Young's modulus of PCL/halloysite composites. Symbols:(■) film casting, (▲) internal mixer, (●) compression molding.

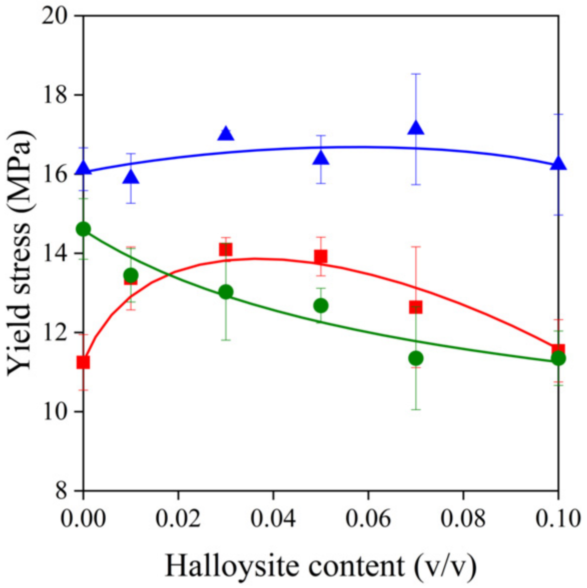

Figure S11. Effect of halloysite content on yield stress of PCL/halloysite composites. Symbols:(■) film casting, (▲) internal mixer, (●) compression molding.

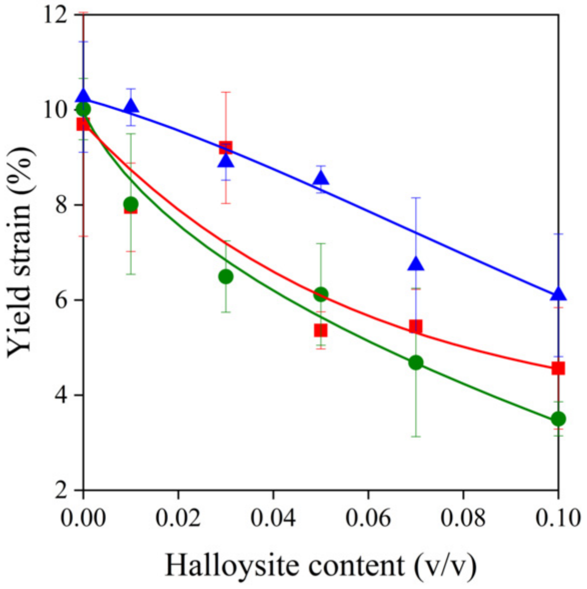

Figure S12. Effect of halloysite content on yield strain of PCL/halloysite composites. Symbols:(■) film casting, (▲) internal mixer, (●) compression molding.

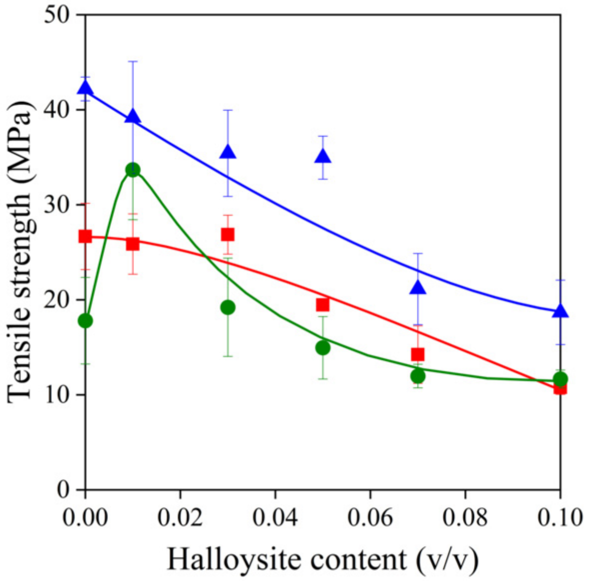

Figure S13. Effect of halloysite content on tensile strength of PCL/halloysite composites. Symbols:(■) film casting, (▲) internal mixer, (●) compression molding.

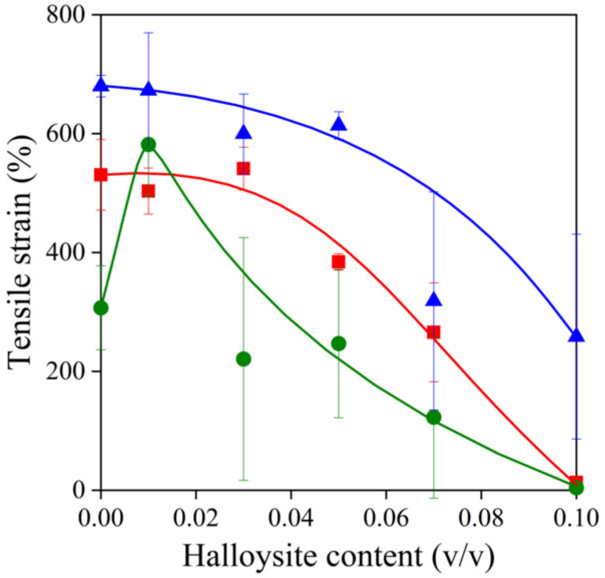

Figure S14. Effect of halloysite content on tensile strain of PCL/halloysite composites. Symbols:(■) film casting, (▲) internal mixer, (●) compression molding.

3. Results of thermogravimetric analysis (TGA)

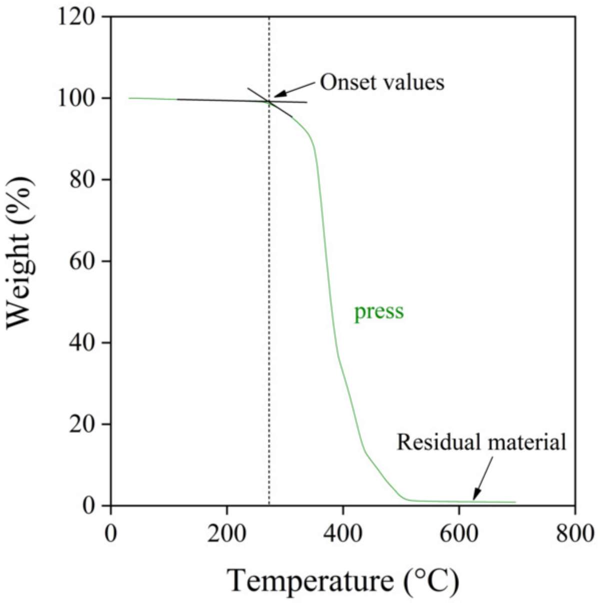

Figure S15. The principle of the determination of onset value. PCL/halloysite composite at 1 vol% halloysite content prepared by compression molding.

Table S5. Results of TGA measurements of PCL/halloysite composites prepared by film casting.

Filler content		Onset, weight (%)	Onset, temperature (°C)	Residual material (%)
Film	(v%)			
0 HAL	0.00	99.95 ± 0.23	266.43 ± 2.10	0.05 ± 0.00
1 HAL	1.00	99.33 ± 0.21	266.48 ± 3.16	1.56 ± 0.48
3 HAL	3.00	97.47 ± 0.25	271.17 ± 3.89	0.57 ± 1.23
5 HAL	5.00	98.03 ± 0.32	270.14 ± 3.41	4.59 ± 1.88
7 HAL	7.00	97.70 ± 0.67	275.68 ± 3.03	5.40 ± 2.56
10 HAL	10.00	97.42 ± 0.81	275.15 ± 4.42	11.02 ± 2.04

Table S6. Results of TGA measurements of PCL/halloysite composites prepared by compression molding.

Filler content		Onset, weight (%)	Onset, temperature (°C)	Residual material (%)
Press	(v%)			
0 HAL	0.00	98.96 ± 0.28	280.26 ± 3.09	0.00 ± 0.00
1 HAL	1.00	99.08 ± 0.07	274.13 ± 3.51	0.99 ± 0.68
3 HAL	3.00	99.34 ± 0.14	270.75 ± 3.83	2.65 ± 1.71
5 HAL	5.00	99.12 ± 0.61	272.42 ± 4.06	7.11 ± 2.66
7 HAL	7.00	99.21 ± 0.93	262.37 ± 3.13	12.83 ± 3.54
10 HAL	10.00	98.88 ± 0.74	265.08 ± 3.99	9.96 ± 2.87

Table S7. Results of TGA measurements of PCL/halloysite composites prepared by internal mixer.

Filler content		Onset, weight (%)	Onset, temperature (°C)	Residual material (%)
IM	(v%)			
0 HAL	0.00	96.12 ± 0.67	263.48 ± 2.31	0.00 ± 0.00
1 HAL	1.00	98.66 ± 0.75	266.25 ± 2.72	0.00 ± 1.32
3 HAL	3.00	98.82 ± 0.93	269.76 ± 3.56	2.62 ± 2.11
5 HAL	5.00	98.10 ± 0.08	272.74 ± 2.43	5.26 ± 1.73
7 HAL	7.00	98.47 ± 0.45	268.78 ± 3.67	8.96 ± 1.16
10 HAL	10.00	98.64 ± 0.83	263.92 ± 3.34	14.09 ± 0.73

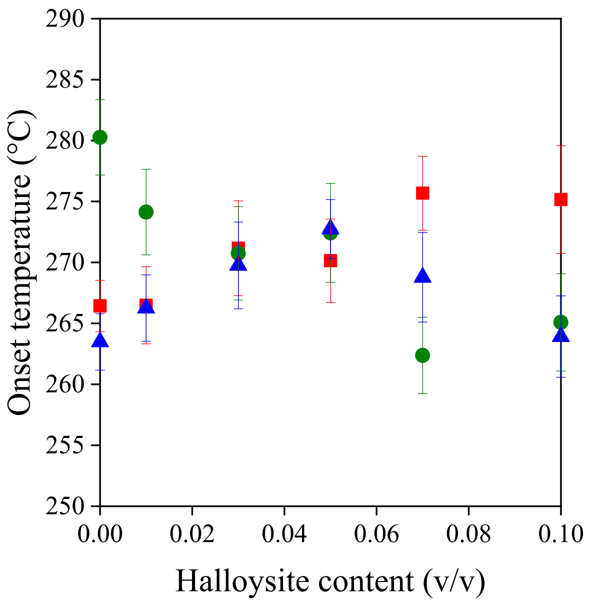

Figure S16. Onset temperature values of PCL/halloysite composites. Symbols:(■) film casting, (▲) internal mixer, (●) compression molding.

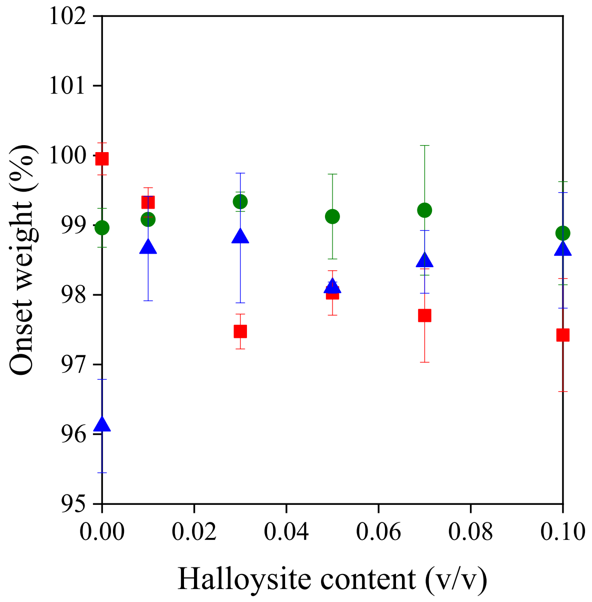

Figure S17. Onset weight values of PCL/halloysite composites. Symbols:(■) film casting, (▲) internal mixer, (●) compression molding.

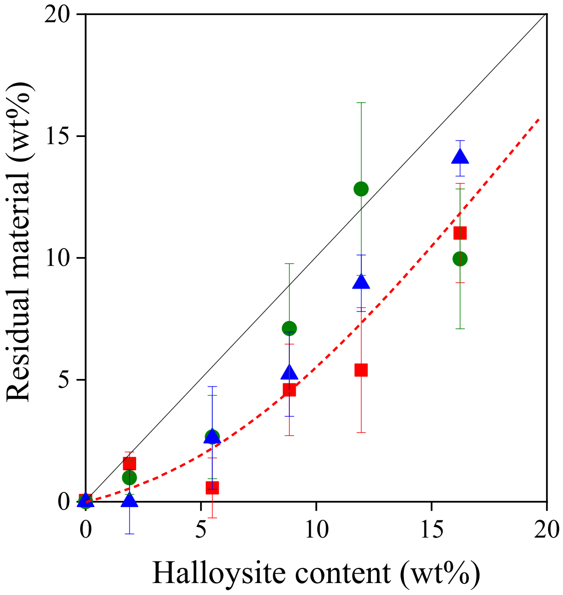

Figure S18. The residual material mass of PCL/halloysite composites as function of filler content. Symbols:(■) film casting, (▲) internal mixer, (●) compression molding.

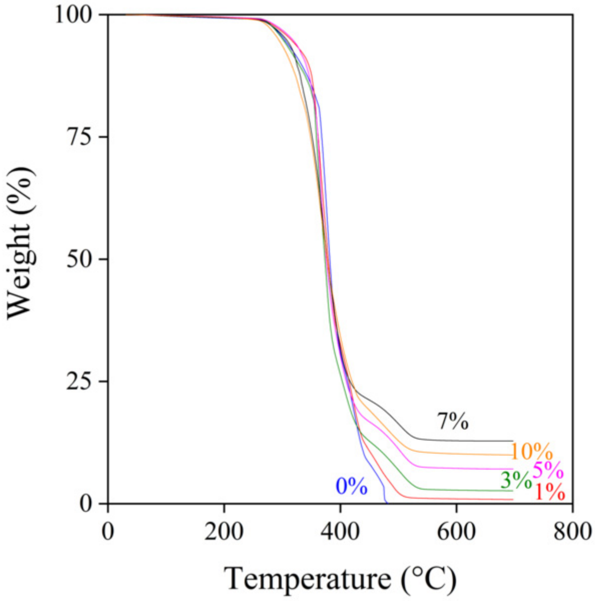

Figure S19. TGA curves of the PCL/halloysite composites with different filler content prepared by compression molding.

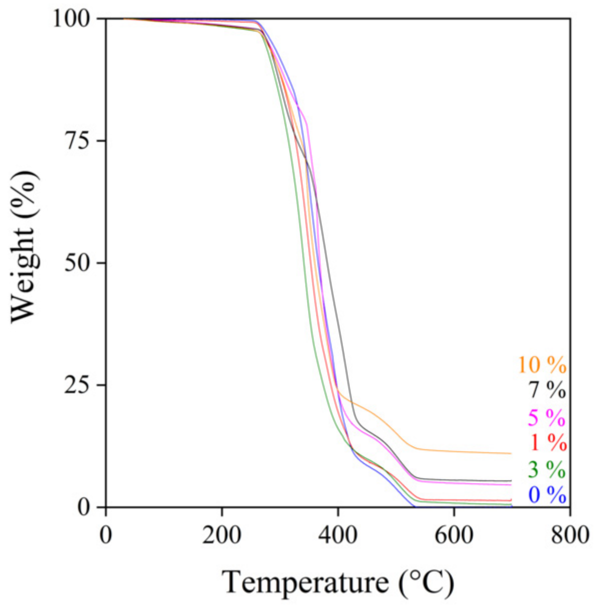

Figure S20. TGA curves of the PCL/halloysite composites with different filler content prepared by film casting.

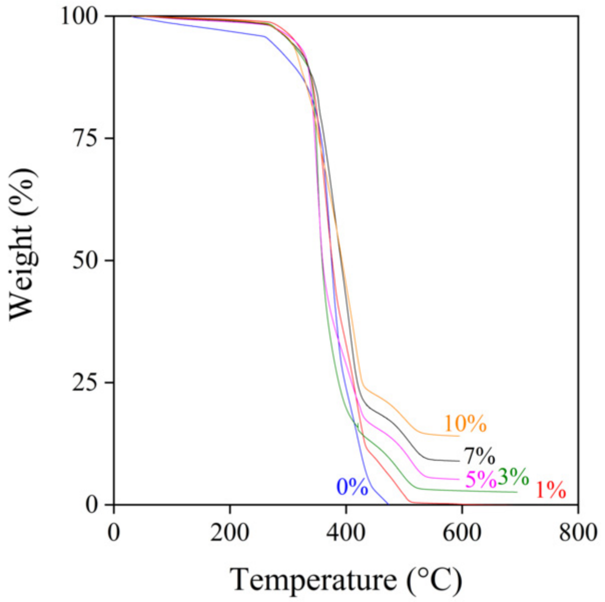

Figure S21. TGA curves of the PCL/halloysite composites with different filler content prepared by internal mixer.

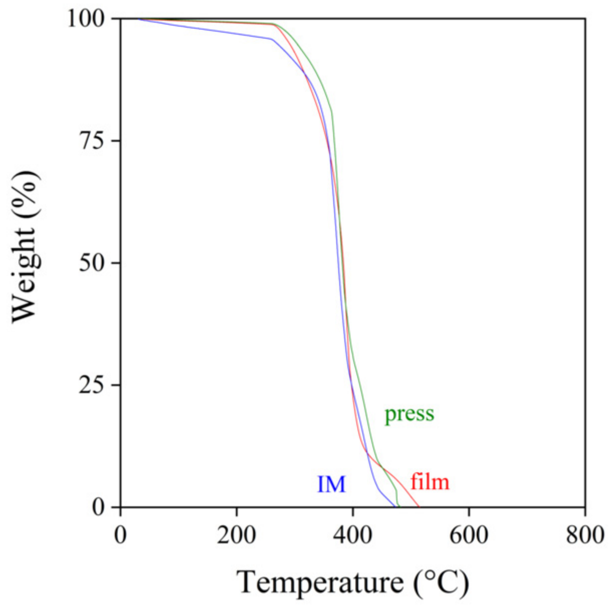

Figure S22. Characteristic TGA curves of the PCL/halloysite composites prepared by different technique at 0 vol% filler content.

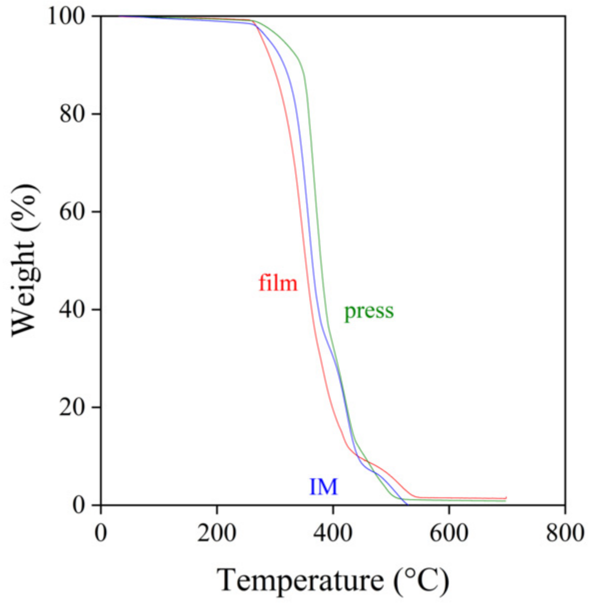

Figure S23. Characteristic TGA curves of the PCL/halloysite composites prepared by different technique at 1 vol% filler content.

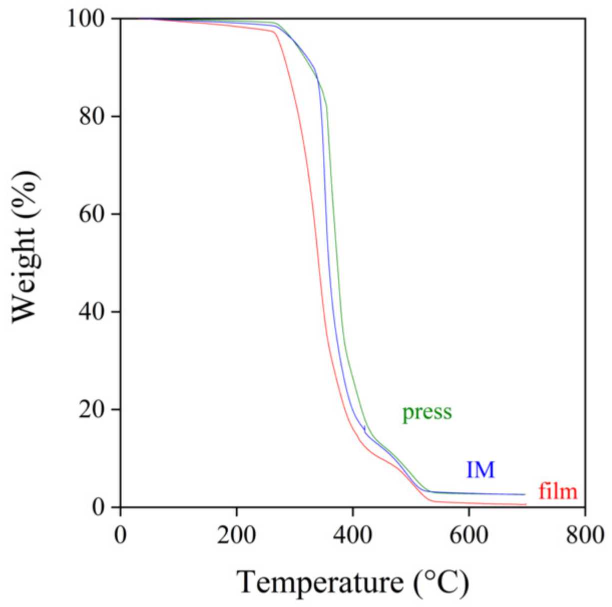

Figure S24. Characteristic TGA curves of the PCL/halloysite composites prepared by different technique at 3 vol% filler content.

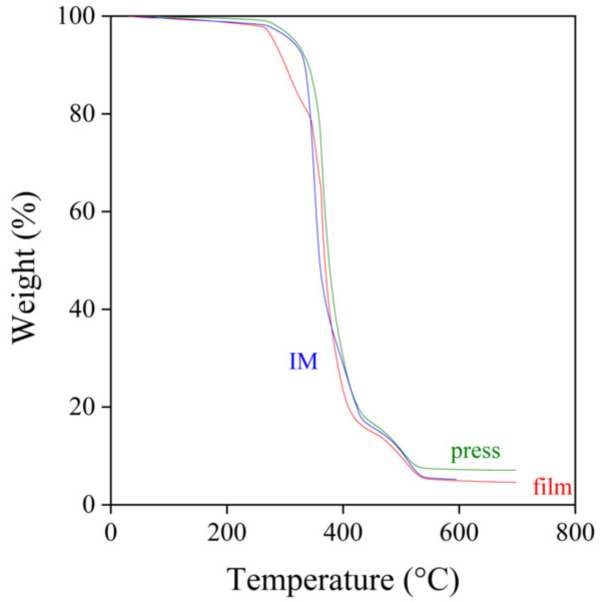

Figure S25. Characteristic TGA curves of the PCL/halloysite composites prepared by different technique at 5 vol% filler content.

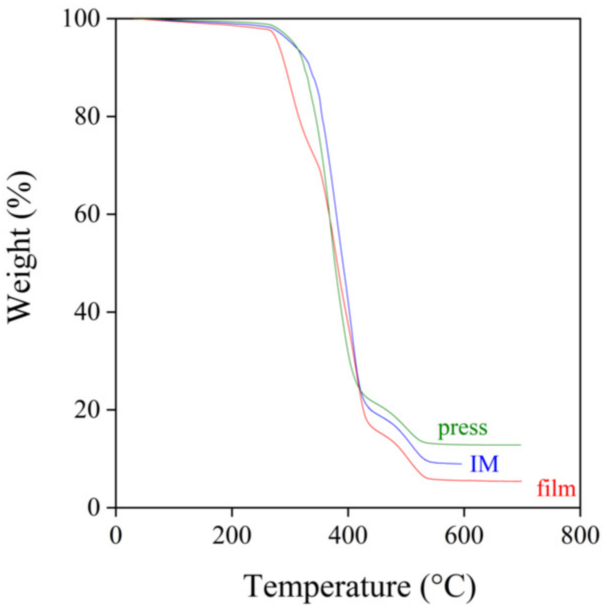

Figure S26. Characteristic TGA curves of the PCL/halloysite composites prepared by different technique at 7 vol% filler content.

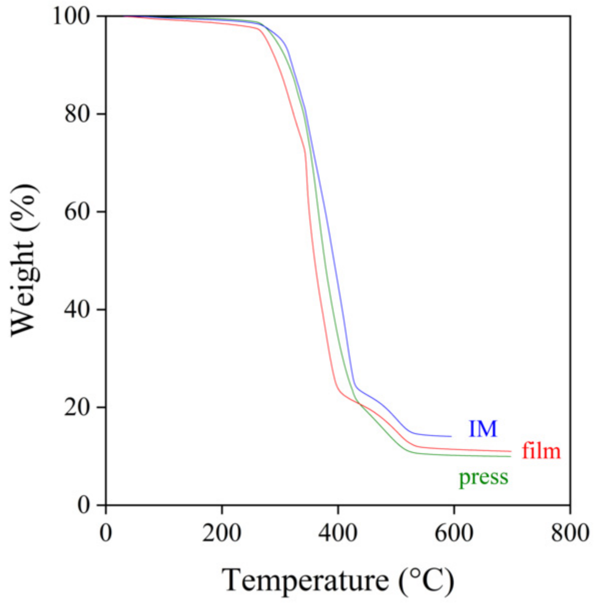

Figure S27. Characteristic TGA curves of the PCL/halloysite composites prepared by different technique at 10 vol% filler content.

4. Results of differential scanning calorimetry (DSC)

Table S8. Results of DSC measurements of PCL/halloysite composites with variant filler content prepared by different technology.

Technology/Sample		Halloysite content (v%)	Cooling		Heating (second heating)		
			T_c (°C)	ΔH_c (J/g)	T_g (°C)	T_m (°C)	ΔH_m (J/g)
**film casting**	**PCL/0Hal**	0	28.19	-70.09	-63.89	54.79	75.72
	PCL/1Hal	1	32.17	-69.76	-61.34	55.61	74.67
	PCL/3Hal	3	29.68	-68.52	-63.45	55.28	73.05
	PCL/5Hal	5	29.36	-69.30	-62.46	54.45	71.13
	PCL/7Hal	7	29.03	-70.81	-61.55	53.95	73.23
	PCL/10Hal	10	31.47	-73.27	-59.24	56.73	77.22
internal mixer	PCL/0Hal	0	30.71	-55.42	-61.07	55.59	64.01
	PCL/1Hal	1	31.40	-68.12	-62.33	56.61	69.70
	PCL/3Hal	3	29.90	-59.69	-61.87	55.27	65.56
	PCL/5Hal	5	30.40	-59.94	-61.95	55.95	64.82
	PCL/7Hal	7	31.68	-56.87	-61.60	57.55	64.46
	PCL/10Hal	10	31.39	-63.23	-	54.28	59.96
compression molding	PCL/0Hal	0	30.40	-61.94	-56.99	54.95	62.47
	PCL/1Hal	1	30.23	-63.66	-62.29	54.29	66.17
	PCL/3Hal	3	30.54	-58.12	-61.25	54.10	61.98
	PCL/5Hal	5	31.55	-63.18	-59.72	55.94	65.74
	PCL/7Hal	7	29.41	-66.27	-61.41	54.29	67.00
	PCL/10Hal	10	31.06	-66.64	-62.61	54.78	71.96

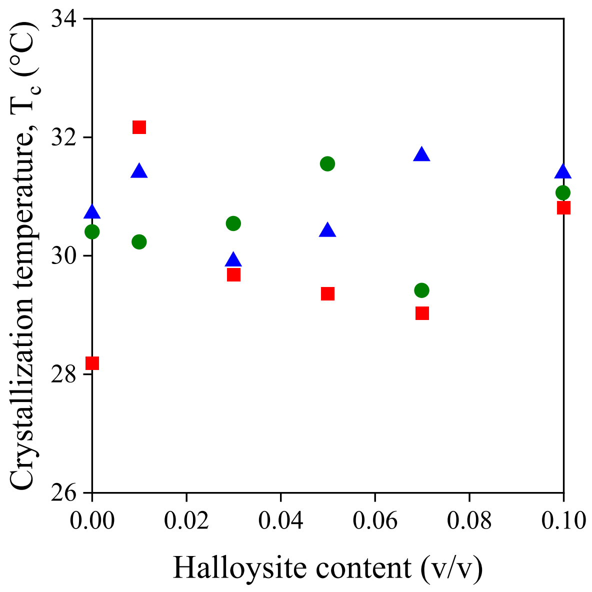

Figure S28. Effect of halloysite content on crystallization temperature of PCL/halloysite composite. Symbols:(■) film casting, (▲) internal mixer, (●) compression molding.

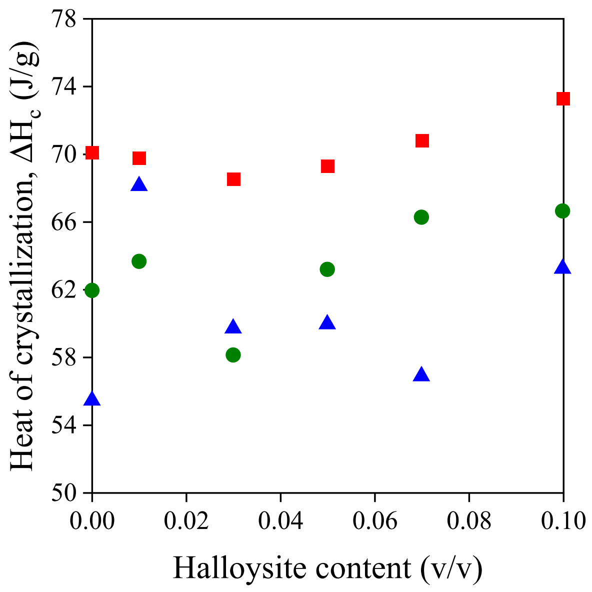

Figure S29. Effect of halloysite content on crystallization enthalpy (ΔH_c) of PCL/halloysite composite. Symbols:(■) film casting, (▲) internal mixer, (●) compression molding.

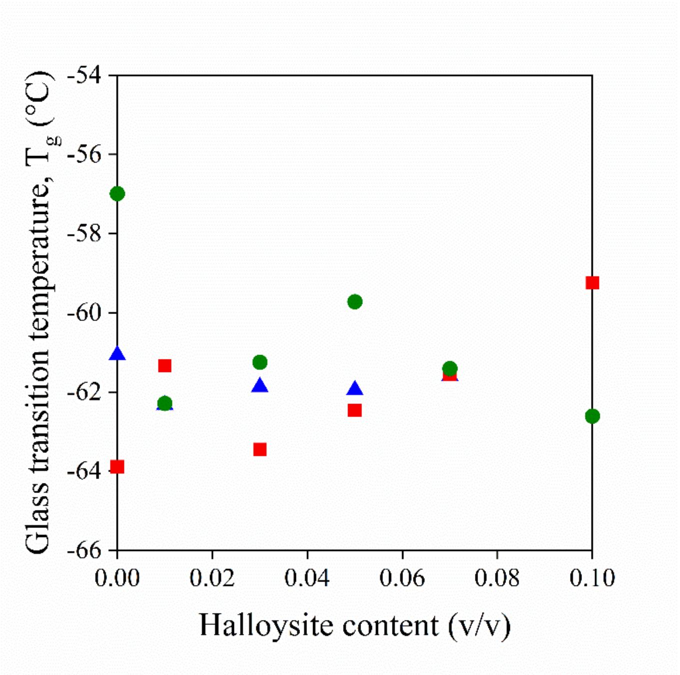

Figure S30. Effect of halloysite content on transition temperature of PCL/halloysite composite derived from second heating. Symbols:(■) film casting, (▲) internal mixer, (●) compression molding.

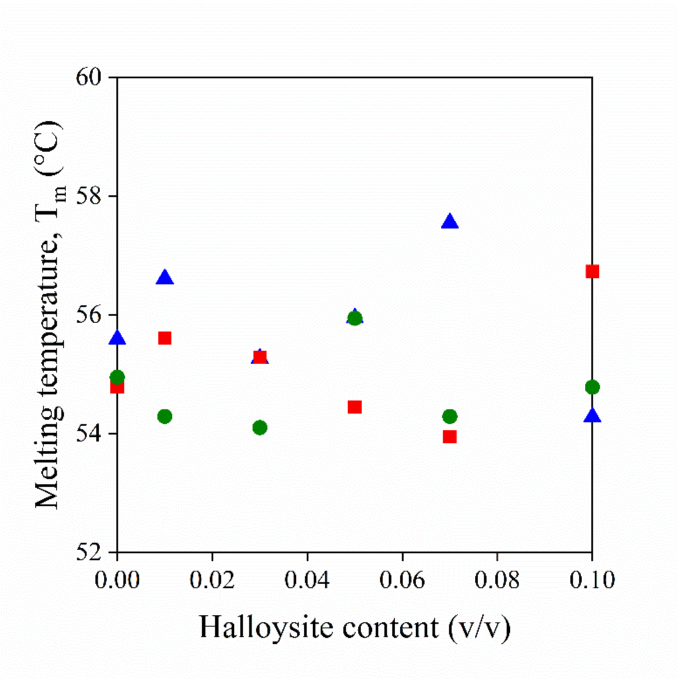

Figure S31. Effect of halloysite content on melting temperature of PCL/halloysite composite derived from second heating. Symbols:(■) film casting, (▲) internal mixer, (●) compression molding.

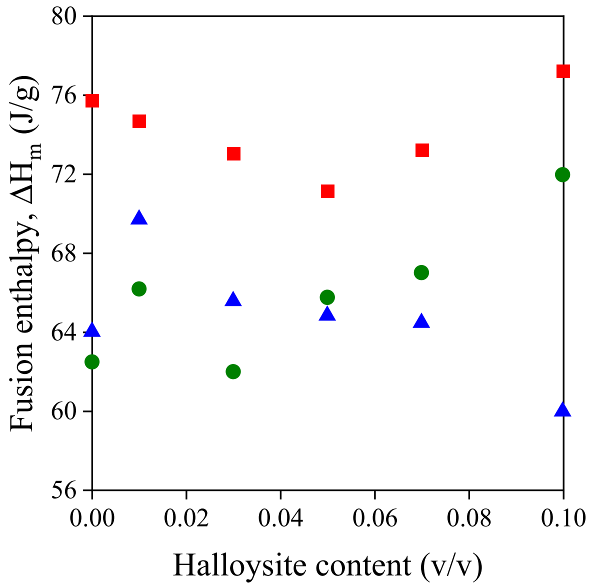

Figure S32. Effect of halloysite content on melting enthalpy (ΔH_m) of PCL/halloysite composite derived from second heating. Symbols:(■) film casting, (▲) internal mixer, (●) compression molding.

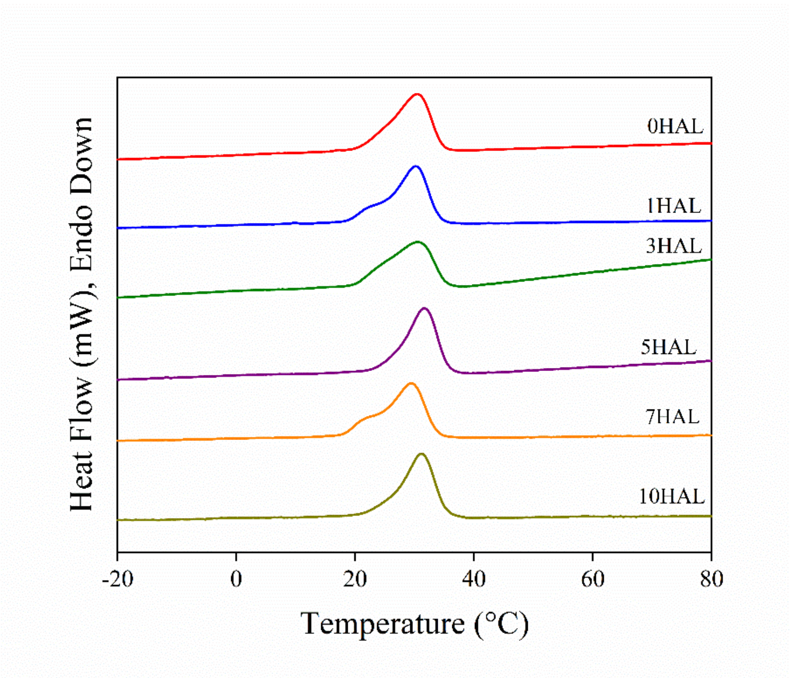

Figure S33. Crystallization curves of PCL/Halloysite composites with different filler content prepared by compression molding.

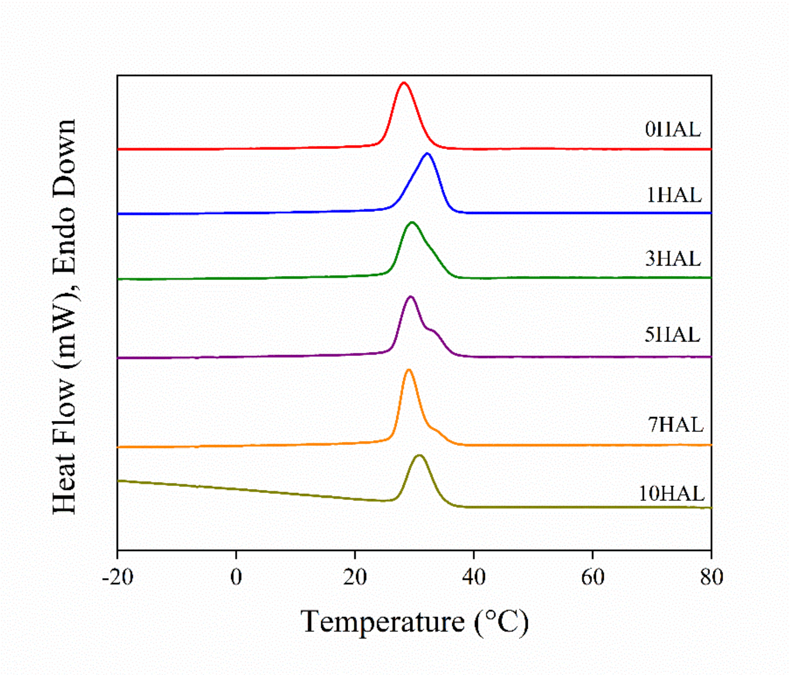

Figure S34. Crystallization curves of PCL/Halloysite composites with different filler content prepared by film casting.

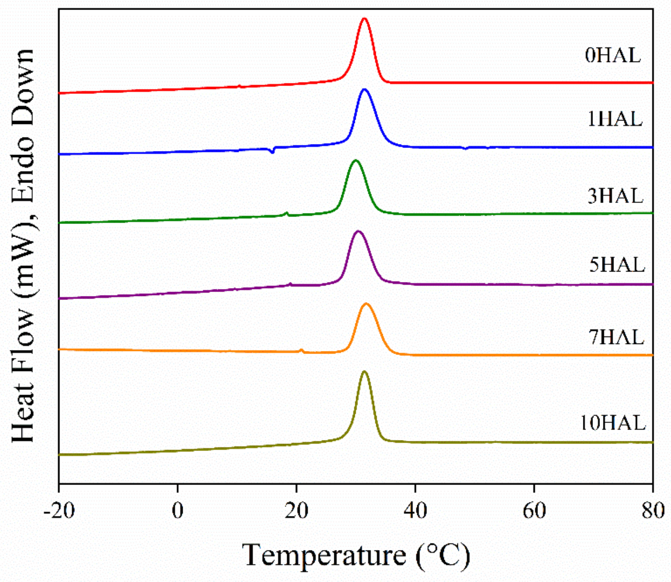

Figure S35. Crystallization curves of PCL/Halloysite composites with different filler content prepared by internal mixer.

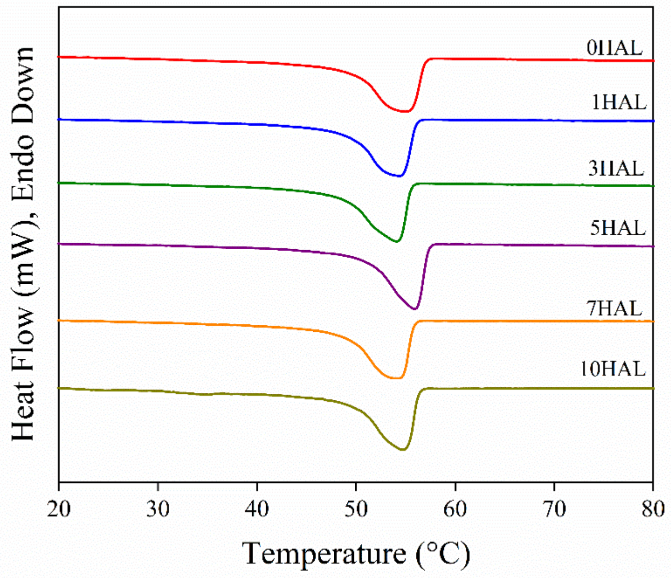

Figure S36. Second heating run of DSC curves of PCL/Halloysite composites with different filler content prepared by compression molding.

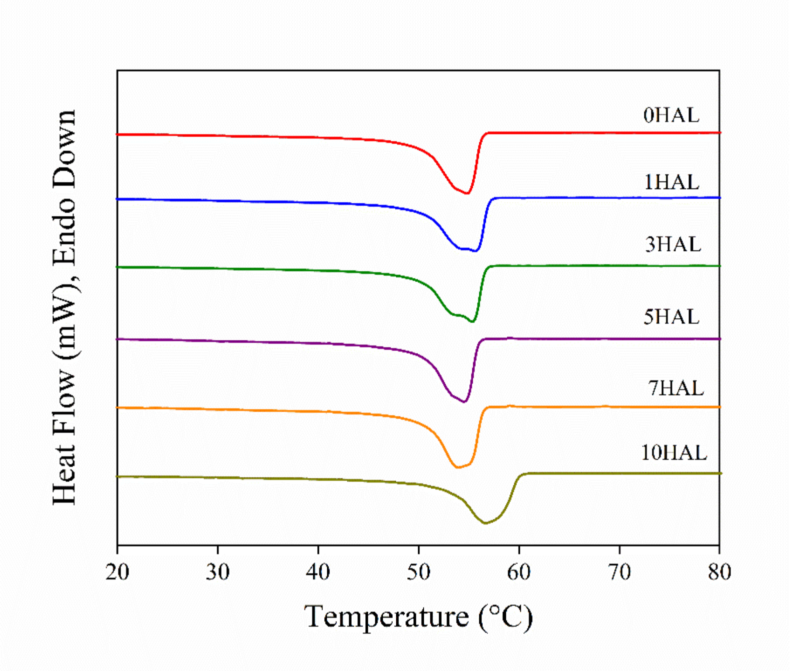

Figure S37. Second heating run of DSC curves of PCL/Halloysite composites with different filler content prepared by film casting.

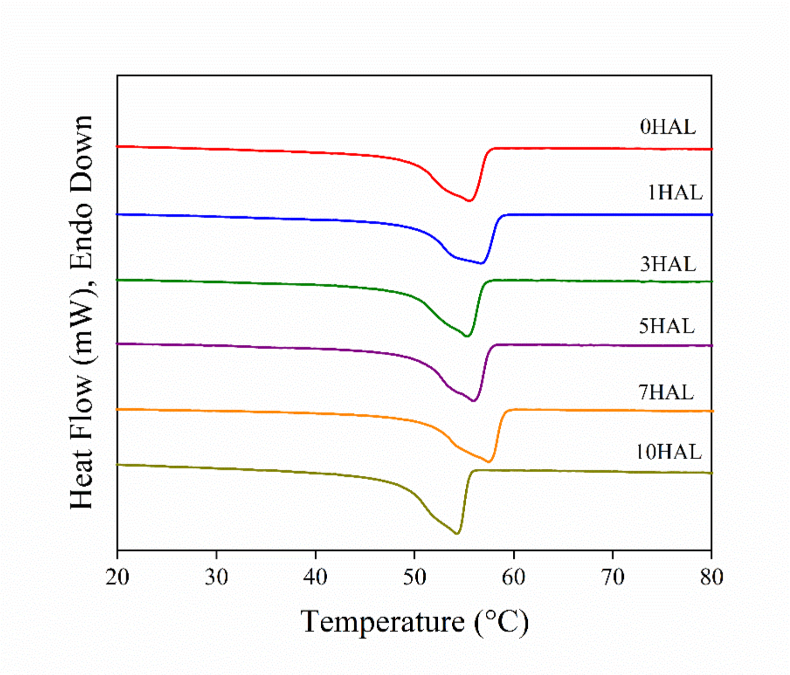

Figure S38. Second heating run of DSC curves of PCL/Halloysite composites with different filler content prepared by internal mixer.

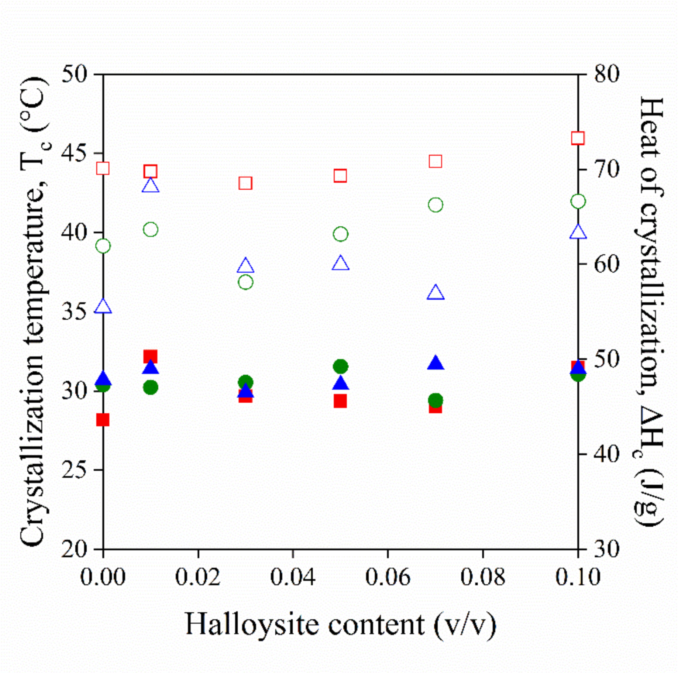

Figure S39. Crystallization temperature (T_c) and crystallization enthalpy (ΔH_c) of PCL/Halloysite composites as a function of halloysite content. Symbols: T_c: (■) film casting, (▲) internal mixer, (●) compression molding, ΔH_c: (□) film casting, (Δ) internal mixer, (○) compression molding.

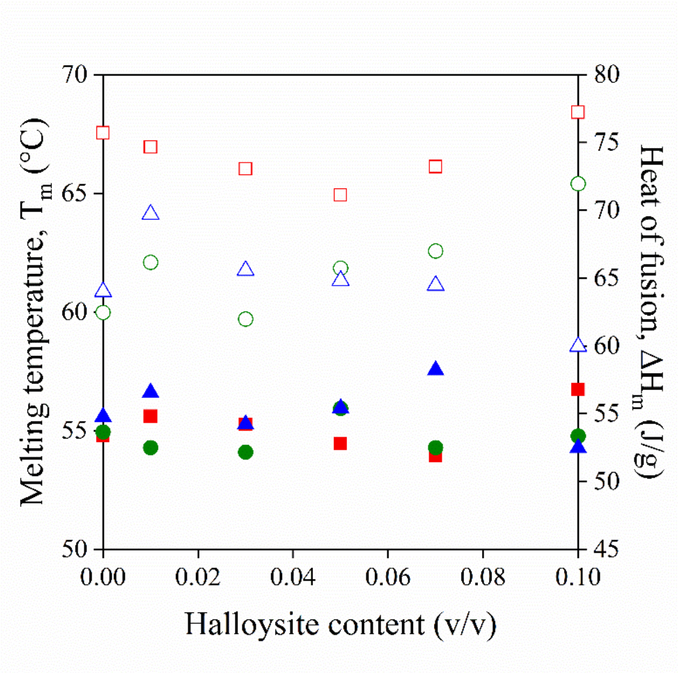

Figure S40. Melting temperature (T_m) and melting enthalpy (ΔH_m) of PCL/Halloysite composites as a function of halloysite content. Symbols: T_c: (■) film casting, (▲) internal mixer, (●) compression molding, ΔH_c: (□) film casting, (Δ) internal mixer, (○) compression molding.

5. Results of dynamical mechanical analysis (DMA)

Table S9. Results of DMA measurements of PCL/halloysite composites prepared by film casting.

	Sample	tan δ (°C)	E'' (°C)
	PCL_0HAL	-46.60	-58.10
	PCL_1HAL	-46.70	-58.40
film casting	PCL_3HAL	-48.90	-57.90
	PCL_5HAL	-47.30	-59.29
	PCL_7HAL	-49.50	-57.10
	PCL_10HAL	-42.70	-54.40

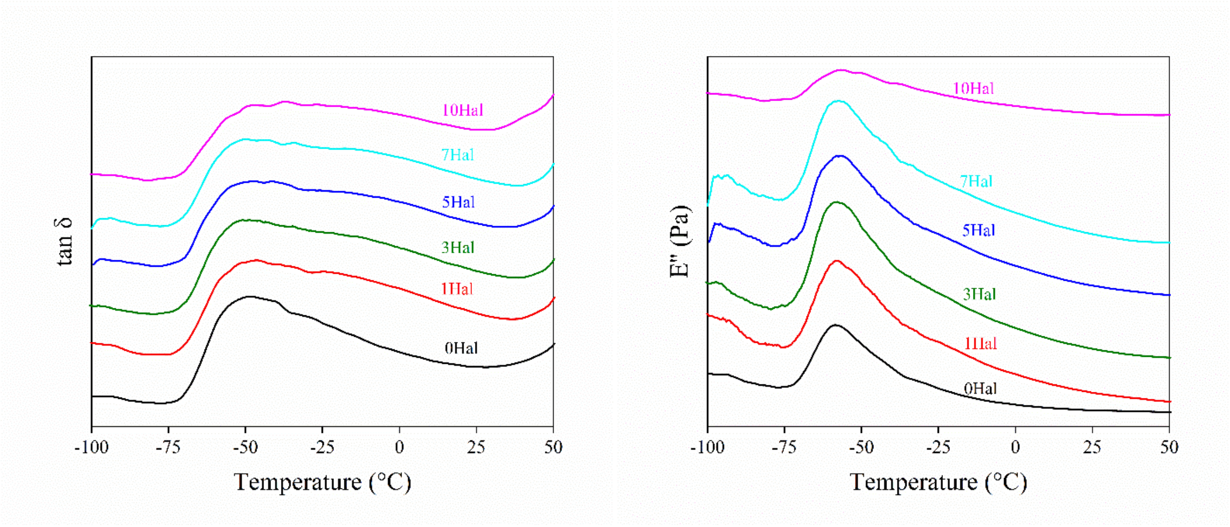

a) b)

Figure S41. Results of DMA measurements of PCL/halloysite composites prepared by film casting. **a**) tan δ, **b**) loss modulus.

Table S10. Results of DMA measurements of PCL/halloysite composites prepared by compression molding.

	Sample	tan δ	E'' (°C)
	**PCL_0HAL**	**-50.70**	**-60.40**
	**PCL_1HAL**	**-50.50**	**-59.70**
**compression molding**	**PCL_3HAL**	**-52.05**	**-59.91**
	**PCL_5HAL**	**-49.60**	**-59.60**
	**PCL_7HAL**	**-49.45**	**-57.73**
	**PCL_10HAL**	**-51.44**	**-59.86**

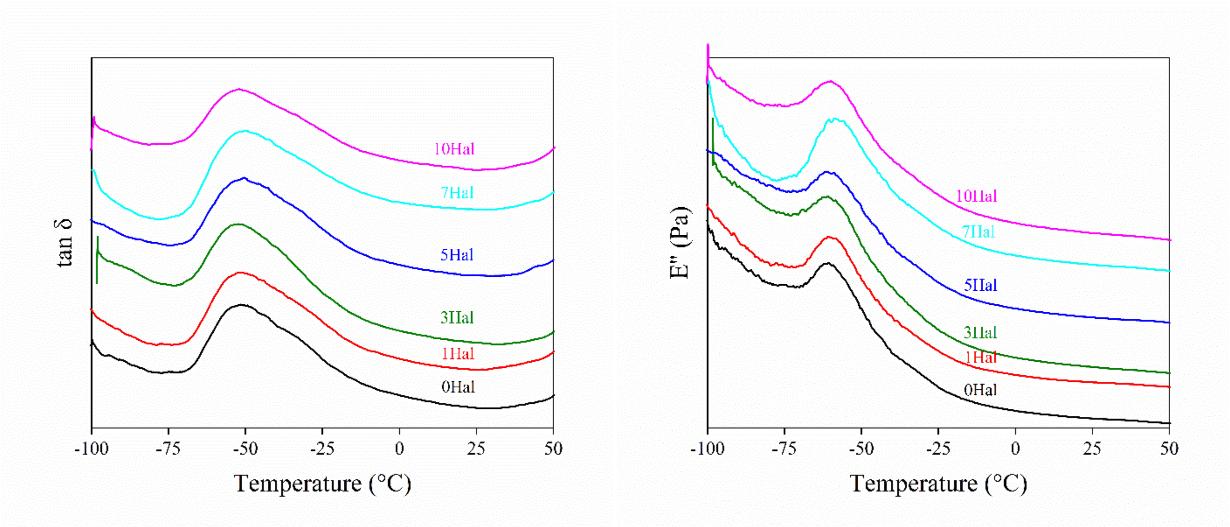

a) b)

Figure S42. Results of DMA measurements of PCL/halloysite composites prepared by compression molding. **a**) tan δ, **b**) loss modulus.

Table S11. Results of DMA measurements of PCL/halloysite composites prepared by internal mixer.

	Sample	tan δ	E'' (°C)
	**PCL_0HAL**	**-50.60**	**-59.60**
	**PCL_1HAL**	**-51.40**	**-59.10**
**internal mixer**	**PCL_3HAL**	**-49.60**	**-57.60**
	**PCL_5HAL**	**-50.00**	**-58.20**
	**PCL_7HAL**	**-50.10**	**-58.10**
	**PCL_10HAL**	**-50.00**	**-58.70**

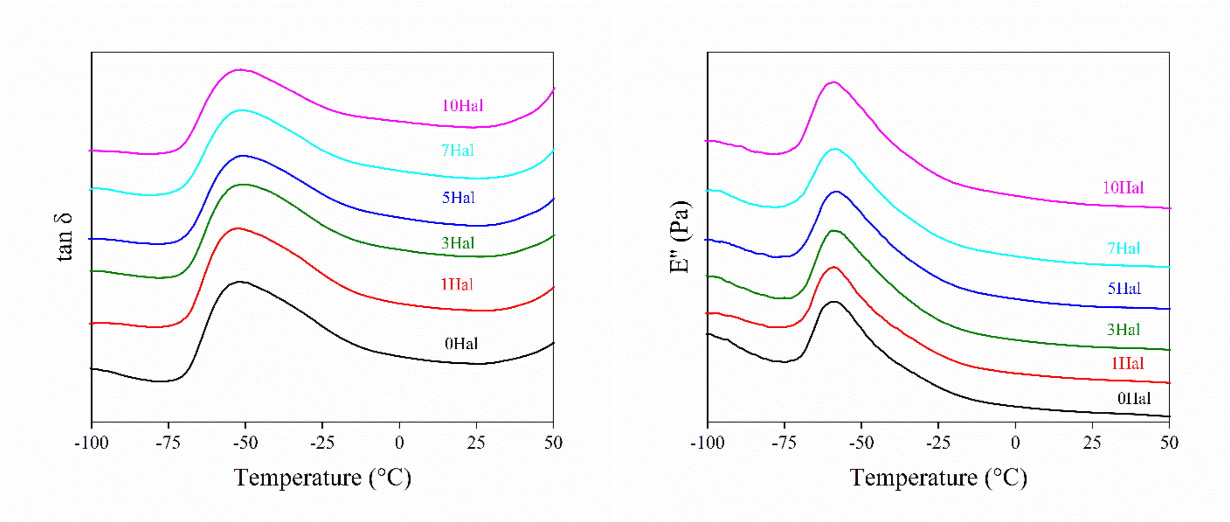

a) b)

Figure S43. Results of DMA measurements of PCL/halloysite composites prepared by internal mixer. **a**) tan δ, **b**) loss modulus.

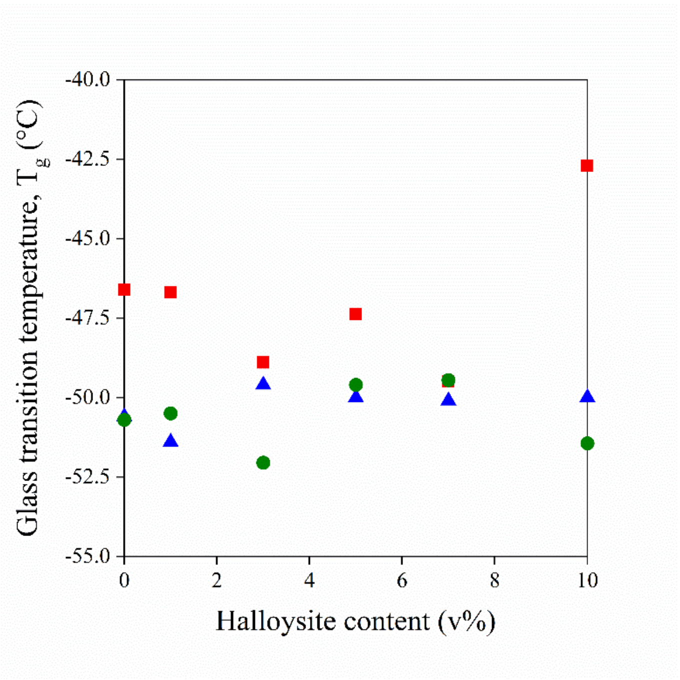

Figure S44. Glass transition temperatures (T_g) derived from tan δ values of PCL/halloysite composites. Symbols: (■) film casting, (▲) internal mixer, (●) compression molding.

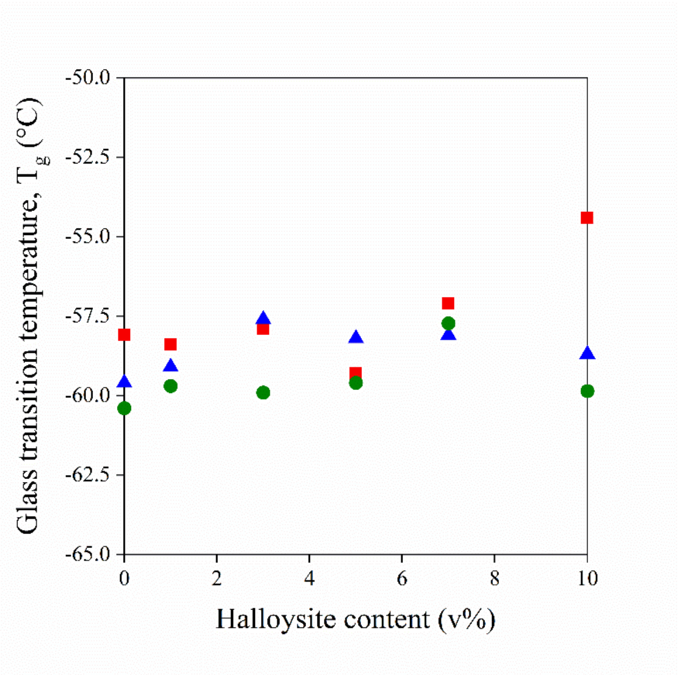

Figure S45. Glass transition temperatures (T_g) derived from E'' values of PCL/halloysite composites. Symbols: (■) film casting, (▲) internal mixer, (●) compression molding.
